# In Situ Neutron Diffraction of Zn-MOF-74 Reveals Nanoconfinement-Induced Effects on Adsorbed Propene

**DOI:** 10.1021/acs.jpcc.3c03225

**Published:** 2023-08-16

**Authors:** Patrick Gäumann, Davide Ferri, Denis Sheptyakov, Jeroen A. van Bokhoven, Przemyslaw Rzepka, Marco Ranocchiari

**Affiliations:** † Laboratory of Catalysis and Sustainable Chemistry, Paul Scherrer Institut, CH-5232 Villigen, Switzerland; ‡ Bioenergy and Catalysis Laboratory, Paul Scherrer Institut, CH-5232 Villigen, Switzerland; § Laboratory for Neutron Scattering and Imaging, Paul Scherrer Institut, CH-5232 Villigen, Switzerland; ∥ Institute of Chemical and Bioengineering, ETH Zurich, CH-8093 Zurich, Switzerland

## Abstract

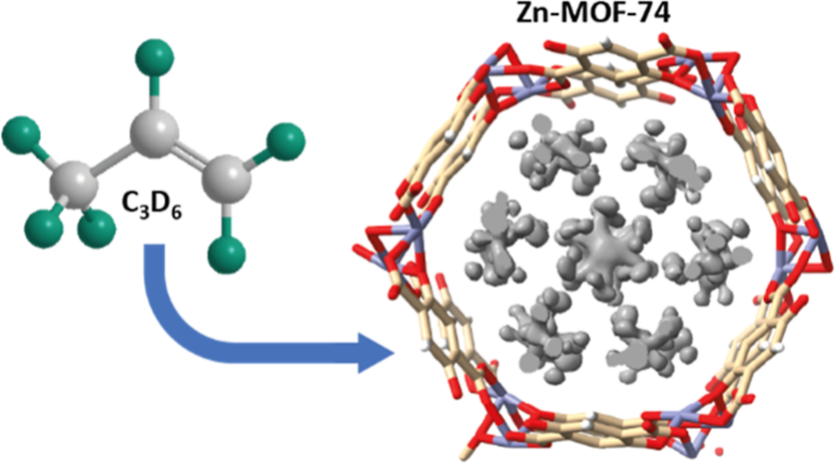

Even though confinement was identified as a common element of selective catalysis and simulations predicted enhanced properties of adsorbates within microporous materials, experimental results on the characterization of the adsorbed phase are still rare. In this study, we provide experimental evidence of the increase of propene density in the channels of Zn-MOF-74 by 16(2)% compared to the liquid phase. The ordered propene molecules adsorbed within the pores of the MOF have been localized by in situ neutron powder diffraction, and the results are supported by adsorption studies. The formation of a second adsorbate layer, paired with nanoconfinement-induced short intermolecular distances, causes the efficient packing of the propene molecules and results in an increase of olefin density.

## Introduction

Metal–organic frameworks (MOFs) are an emerging class of porous materials consisting of metal ions or clusters connected by organic linkers.^1^ Their modular construction enables the precise tuning of properties such as pore diameter and surface area and the introduction of various functional groups by varying the size and functionality of linker molecules. Additionally, different metals lead to various coordination modes and thus different (pore) structures.^2−4^ Notably, their flexible structures exhibit high surface areas and allow their application in catalysis,^5−8^ gas storage,^9−12^ and separation.^13−18^ The separation of olefins from paraffins is one of the most important industrial processes^19^ since light olefins are essential feedstocks for the production of synthetic fibers and plastics, as well as chemicals like acetic acid and acetone.^20^ Every year, more than 200 million tons of ethene and propene are produced by cryogenic distillation, a very energy- and capital-cost-intensive process.^19,21^ The separation of alkenes from alkanes by MOFs relies on specific interactions between the π-system of the former and the porous framework, namely its open metal sites (OMS) and functional groups.^11,16−18,22−25^ M-MOF-74 (M = Mg, Mn, Fe, Co, Ni, Cu, and Zn) are promising candidates for the separation of alkenes from alkanes due to the high density of OMS (7.13–7.58 mmol M^2+^ per cm^3^).^18,25^ The framework consists of metal nodes coordinated to three carboxyl and two hydroxyl groups, forming helical rods of edge-sharing square pyramids. These rods are connected by the benzene ring of the 2,5-dioxidoterephthalate linker to form 10 Å wide hexagonal 1D channels arranged in a honeycomb-like structure.^26,27^ The positions of propene within M-MOF-74 were investigated before by neutron powder diffraction (NPD). Distances of 2.60(2) and 2.56(2) Å were reported between the OMS in Fe-MOF-74 and propene C_1_ and C_2_, respectively.^17^ The equivalent distances in Co-MOF-74 were determined as 2.66(5) and 2.73(6) Å.^16^

MOFs are extensively applied in catalysis, creating an environment that might not be achievable in homogeneous or typical heterogeneous systems. This class of materials influences the reaction environment and thereby changes the product distribution, allows alternative reaction mechanisms, and promotes reactions by the adsorption of substrates.^28,29^ The selective adsorption on microporous materials can influence the catalytic reaction rates and equilibrium yields by changing the potential energy surface.^30^ For example, the interface between silver nanoparticles and a ZIF-8 layer allowed performing a Kolbe–Schmitt reaction at 1 bar of CO_2_ and room temperature.^31^ This was achieved by the high CO_2_ concentration accumulated by adsorption on the framework. The regioselectivity was inverted compared to the usually applied reaction conditions (>125 °C and >80 bar CO_2_). In a different system, the adsorption of CO_2_ by a Cd-MOF allowed the carboxylative cyclization of multiple propargylamines at 5 bar and 60 °C.^32^ Another Zn-based MOF allowed the cycloaddition of propylene oxides to yield propylene carbonates at 1 bar CO_2_ and 100 °C, due to its high affinity to CO_2_.^33^ Furthermore, two Zn-MOFs of MOF-5 and UMCM-1 topology inverted the reactive character of a phosphonium zwitterion to an electrophile. This yielded the Aldol–Tischenko instead of the expected Morita–Baylis–Hillman product.^34^ Our group discovered that the addition of Zn-MOF-74 in the Co-catalyzed hydroformylation of linear terminal olefins significantly enhances the selectivity toward the branched aldehydes.^35^ Monte Carlo simulations combined with kinetic modeling disclosed that the high alkene density within the MOF pores favored the formation of the *iso* products. The above examples demonstrate the influence of nanoconfinement effects, such as restricted space within the pores or preferential adsorption, on the adsorbate phase. Under nanoconfinement, the adsorbed phase exhibits distinctly different properties, behavior, and reactivity than the liquid or gas phases, offering potential advantages in adsorption, separation, and catalysis. The modulated local concentrations within the pores of microporous materials can influence the product distribution and the substrate reactivity. There is little experimental characterization of nanoconfined species, which is required to understand the origin of the divergent chemical and physical properties. These experimental data are required to further advance our understanding of nanoconfinement effects and to confirm the simulation results. In this study, we determined the structure of propene within the channels of Zn-MOF-74 by in situ NPD, thereby rationalizing its increased density.

## Methods

All solvents and chemicals were purchased from commercial suppliers and used without further purification unless otherwise specified. 2,5-Dihydroxyterephthalic acid was recrystallized from ethanol/water (1:1).

Zn-MOF-74 was synthesized using the following procedure: 2,5-dihydroxyterephthalic acid (1.80 g, 9.08 mmol, 1.00 equiv) and zinc(II) acetylacetonate monohydrate (5.20 g, 18.5 mmol, 2.00 equiv) were dissolved in dimethylformamide (DMF, 176 mL) and water (9 mL). The solution was split equally between three EasyPrep vessels (100 mL), which were placed in a Mars5 CEM microwave reactor heated to 403 K (20 min ramp) for 1 h, yielding a yellow suspension. The solid was filtered off and washed with DMF (3 × 100 mL), ethanol (3 × 100 mL), and *tert*-butylmethylether (3 × 100 mL). The yellow powder was then boiled in methanol overnight, filtered off, and dried in a vacuum oven at 333 K for 2 h. The solid was activated at 523 K in a vacuum overnight to yield the yellow product (2.22 g, 75%).

Powder X-ray diffraction was measured in Bragg–Brentano geometry using an in-house Bruker D8 Advance diffractometer equipped with a Lynxeye XE detector. Monochromatic X-ray radiation of λ = 1.542 Å, generated by a 2.2 kW Cu anode long fine focus ceramic X-ray tube operated at *V* = 40 kV and *I* = 40 mA, was used. The data were recorded with 0.02° steps in the range of 4–40° 2θ.

Nitrogen adsorption experiments were performed on a Micromeritics 3Δ Flex surface characterization instrument. The sample was equilibrated against doses of N_2_ in the range of 0–1 bar at 77 K. Zn-MOF-74 was activated at 523 K for 20–24 h under vacuum on a Micromeritics VacPrep 061 sample degas system before measurements. The gravimetric surface area was calculated using the Brunauer–Emmett–Teller (BET) method after fitting the isotherm data in agreement with the consistency criteria.^36^ Propene physisorption experiments were conducted on the same instrument at around 215 K using a cold bath (dry ice in methanol water 60:40).

Temperature-programmed desorption (TPD) experiments were carried out on a Micromeritics AutoChem II 2920 chemisorption analyzer. Helium was used as the carrier gas. Approximately 100 mg of Zn-MOF-74 was loaded and activated directly by a heat treatment in helium at 523 K for 30 min. Propene was dosed through a loop (100 μL) up to 20 times or until the saturation of the material occurred. The same sample was used for all four temperature ramps, followed by a repetition of the first ramp to ensure the stability of the framework under the used conditions. The shape of the propene desorption curves was comparable between the first ramp and its repetition, indicating that Zn-MOF-74 remained stable throughout the experiments.

Infrared spectroscopy experiments in the attenuated total reflection mode (ATR-IR) were conducted using a spectrometer (Vertex70, Bruker) equipped with a liquid nitrogen-cooled HgCdTe detector and a custom-made flow cell. Spectra were acquired by accumulating 100 scans at a spectral resolution of 4 cm^–1^ and a scanner velocity of 10 kHz. The sample was prepared by loading a suspension of Zn-MOF-74 (ca. 5 mg) in ethanol (2 mL) on a trapezoidal ZnSe crystal (52 × 50 × 20 mm, Crystran) and allowing solvent evaporation. The spectrum of dissolved propene was obtained on the clean ZnSe crystal from the measurement of a saturated solution of propene in cyclohexane against the background of cyclohexane. Liquids were provided to the cell using an HPLC pump (Knauer Azura P4.1S). In the gas-phase experiments, argon was used to dry the MOF layer deposited on the ZnSe crystal as above, and then propene was added to the flow. Prior to the admittance of propene, a background spectrum of the dry MOF layer was collected in argon. Argon and 5 vol % propene in argon were admitted to the cell using calibrated mass flow meters (Bronkhorst) with a flow rate of 40 cm^3^/min.

A series of constant-wavelength NPD data were collected during in situ experiments conducted at the HRPT beamline at the SINQ facility, Paul Scherrer Institut,^37^ using a dedicated sample environment. Zn-MOF-74 was activated in a Schlenk tube in a vacuum at 523 K overnight before the experiment. In a nitrogen-filled glovebox, the sample (786 mg) was placed in a vanadium container (10 × 50 mm), sealed with indium wire, and then connected to a sample holder under ambient atmosphere. The connection through a stainless steel capillary to the gas rig enabled evacuation and the dosing of propene at the selected temperature. Zn-MOF-74 was further activated overnight in a cryofurnace at 453 K in vacuum. Then, it was cooled to 226 K, and deuterated propene (Sigma-Aldrich, volumes corresponding to 1, 2, 4, and 6 mmol/g) was dosed by multiple additions of a known gas volume (1.05 mL, tube plus dead volume of valves) at 1 bar until the desired propene loading was reached. The amount of propene was calculated by the ideal gas law. In the last step, Zn-MOF-74 was saturated at 1 bar of propene (corresponding to 8 mmol/g loading), and data at 226 K were collected. The gas was dosed at 226 K to reach conditions close to the condensation of propene, where the number of adsorbed molecules is maximal. H_2_^38−40^ and CO_2_^41^ were already dosed at temperatures below 240 K, such that no issues with disorder were anticipated. Finally, the saturated sample was evacuated for 30 s to remove the excess of weakly bound molecules, which would crystallize upon cooling, and cooled to 1 K for data collection. Each step was scheduled for 8 h and monitored by a series of NPD scans collected at a wavelength of 1.8857 Å and registered in the range 5–160° 2θ.

Vanadium containers provided the required data quality for the Rietveld analysis. The seven acquired datasets corresponded to the activated sample (Zn-MOF-74-0), five samples with various loadings of propene at 226 K (Zn-MOF-74-1, -2, -4, -6, and -8, corresponding to the propene loadings given in mmol/g), and one sample subjected to partial desorption after saturation with 8 mmol/g and cooled to 1 K (Zn-MOF-74-8_1K). The investigated diffractograms resulted from averaging all scans after attaining equilibrium at each experimental step, indicated by the stability of the relative intensities. The baseline for each dataset was defined using the program lines.^42^ The simulated annealing algorithm implemented in Topas 7^43^ was used to locate the propene molecules within Zn-MOF-74. The model of the framework was refined before in the space group *R*3̅ against Zn-MOF-74-0 data.

Simulated annealing is a global optimization algorithm, particularly well suited for locating organic species inside porous materials.^44^ Four propene molecules, defined as rigid bodies, were placed randomly in the unit cell and moved around by changing their positions, orientations, and free torsion angles. After each rearrangement, the difference between the experimental data of Zn-MOF-74-8 and the calculated profile was determined. Contrary to the Rietveld refinement, the cycle continued after convergence had been reached such that the global minimum could be probed.^45^ The output model was used for the Rietveld refinement of the Zn-MOF-74-8 dataset. The same protocol was adopted for the remaining datasets. If the occupancy of a specific site converged to null, the site was removed. In the datasets collected at 226 K, the propene molecules were defined as rigid bodies throughout the simulated annealing and refinement, whereas for the data measured at 1 K, the bond lengths of the propene molecules were refined. The same instrumental function was refined against all collected data. The peak shape was fitted with the pseudo-Voigt function.

## Results and Discussion

The X-ray diffractogram of Zn-MOF-74 (Figure S1a) revealed exclusively Bragg reflections corresponding to the desired MOF phase, confirming the sample purity. The BET surface area (Figure S1b) and the pore volume determined by nitrogen physisorption were 1290 m^2^/g and 0.459 cm^3^/g, respectively, in good agreement with the literature.^46,47^

**Figure 1:**
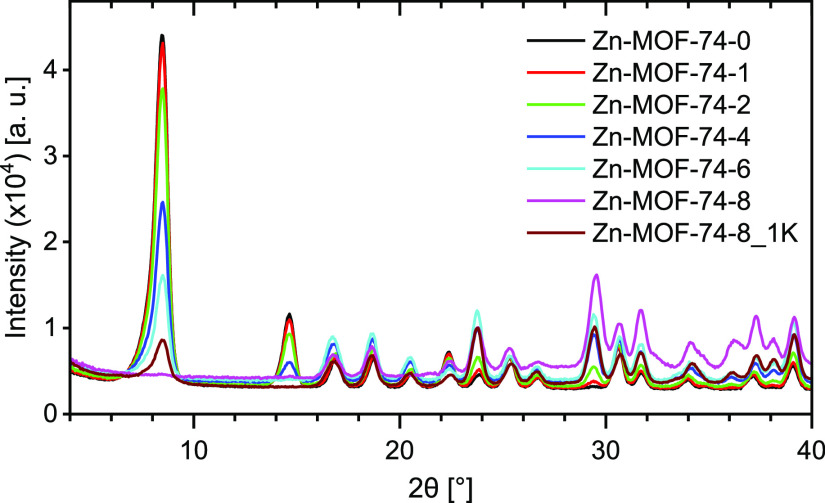
Overview of the NPD patterns at the low 2θ range of Zn-MOF-74 charged with various amounts of deuterated propene. The patterns were collected at 226 K except for Zn-MOF-74-8_1K, which was obtained after the evacuation of the saturated sample by applying vacuum for 30 s and measured at 1 K.

**Figure 2:**
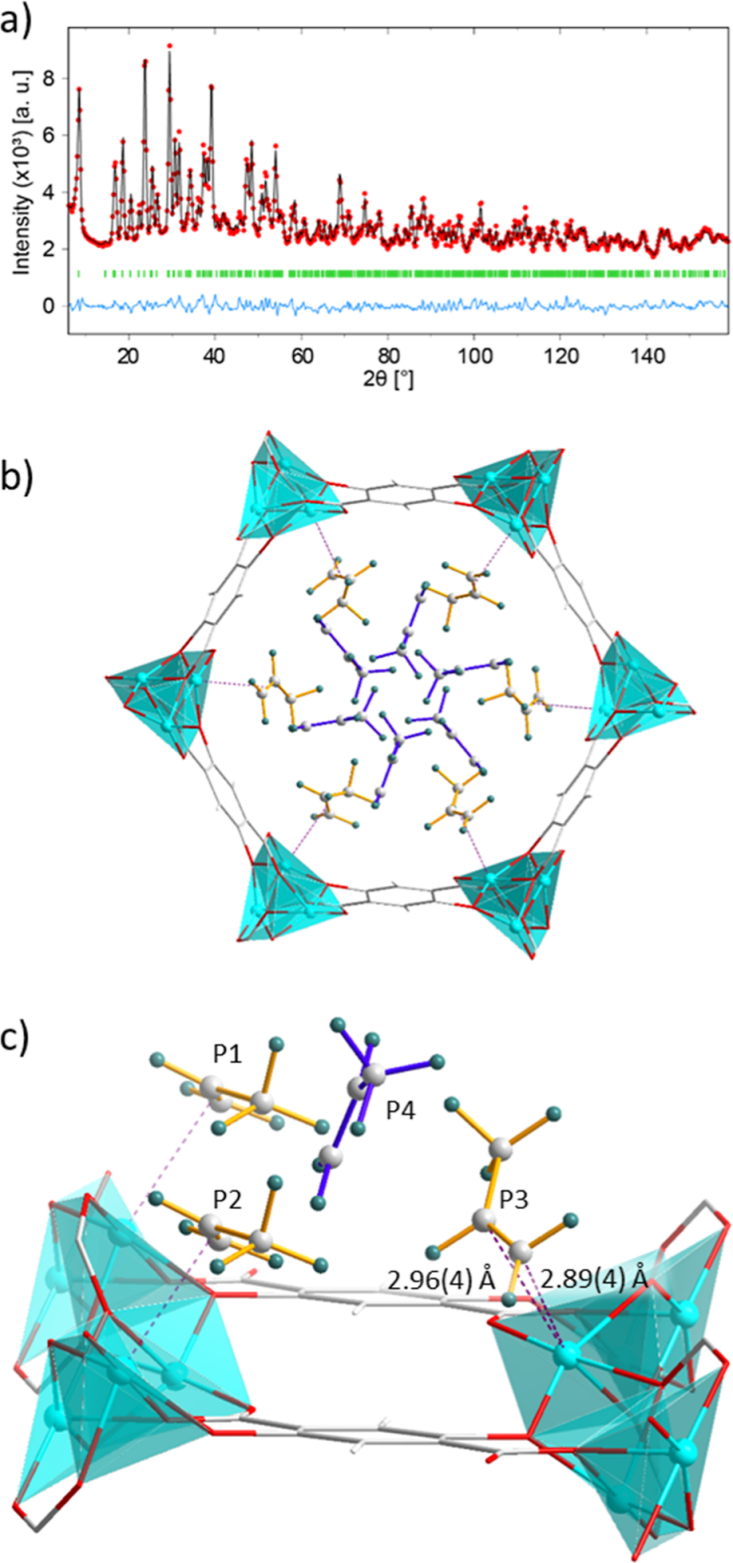
In the Rietveld refinement (a) of Zn-MOF-8_1K, the light blue line represents the difference between the experimental data (red) and the model (black), while peak ticks are indicated in green. In the resulting structure (b) and the local structure in a fraction of the pore (c), propene molecules of the first adsorption layer (P1, P2, and P3) are drawn with yellow bonds, while second-layer propene molecules (P4) feature blue ones. Adsorption of propene molecules to Zn(II) sites (cyan spheres) is depicted by dashed purple lines, while the distances Zn–C_1_ and Zn–C_2_ are represented in a darker shade of purple. Color code: gray, C; white, H; green, D; red, O; and cyan, Zn.

NPD experiments were conducted on Zn-MOF-74, charged with various amounts of deuterated propene (0, 1, 2, 4, 6, and 8 mmol/g of MOF) at 226 K, yielding samples Zn-MOF-74-0 (i.e., activated material), -1, -2, -4, -6, and -8, and -8_1K. Data of Zn-MOF-74-8_1K were registered after the rapid desorption of excess propene from the saturated MOF (Zn-MOF-74-8) and subsequent cooling to 1 K. The refined models of all samples converged with GoF ≈ 1.9–3.4, enabling the pinpointing of the olefin molecules inside Zn-MOF-74. Figure 1 gives an overview of the evolution of Bragg reflections across the collected patterns. The propene loading is strongly reflected by the changes in relative intensities of the first two peaks at 8.5 and 14.6° 2θ, respectively. The intensities diminish with increased dosed amounts, which is indicative of pore filling of porous materials.^48−50^Figures 2a and S2 display the difference between the observed and calculated data. The refined structure of Zn-MOF-74-8 revealed three distinct positions of propene molecules coordinated to the Zn site (Figures S3–S6). An additional molecule points to the pore center, forming a second adsorbate layer (Figure S7). We assume that a maximum of one propene molecule is distributed over the three equivalent positions in the plane perpendicular to the *c* direction based on grand canonical Monte Carlo (GCMC) simulations.^51,52^ Thus, the maximum occupancy of the second adsorption layer sites is one-third. A second coordination layer was already reported for CO_2_^53^ and ethene^16^ adsorbed on MOF-74, while the occupation of secondary sites in propene adsorption on Co-MOF-74^51^ and Mg-MOF-74^52^ was simulated by GCMC. To our knowledge, this is the first experimental observation of a second ordered adsorption layer of propene in the pore space of any M-MOF-74 channel.

Each Zn ion has the ability to coordinate one propene molecule. The fractional occupancies of positions 1, 2, and 3 in the first adsorption layer of Zn-MOF-74-8 are 0.351(12), 0.322(12), and 0.297(15), respectively. Thus, together, the three positions nearly saturate the Zn OMS, while the second adsorption layer site’s occupancy is 0.280(5). This value is close to the maximum occupancy of the second adsorption layer, so Zn-MOF-74 is almost saturated under the applied conditions.

The molecules’ conformations in the populated sites as a function of gas loading are depicted in Figures S3 and S8–S11, whereas Figure S12 shows the respective isotherms. The occupancy of primary adsorption sites rapidly develops with the increasing dosage. We assume that the second adsorption layer can be populated exclusively after the sufficient occupation of the OMS to allow adequate intermolecular interactions. The saturation of the OMS corresponds to a loading of 6.16 mmol/g such that the first occurrence of the second layer in Zn-MOF-74-6 seems reasonable. The data of Zn-MOF-74-4 are indicative of a more dynamic behavior of the first adsorbate layer, as reflected by the enhanced displacement parameters that needed to be restrained. As a result, the corresponding structure model is less robust, and the refined occupancies are less reliable (Figure S13). They should not be compared with the models under all other conditions.

In addition to these two adsorption layers, there are disordered positions of propene in the channel center. Adding these molecules to the structural model significantly reduces the *R* factors, as expected from the central electron density in Zn-MOF-74-2 (Figure S17b). Even though these molecules are located in the center of the pore as well, they occupy a different crystallographic position compared to the molecules of the second adsorption layer. They do not adopt a defined structure and are identified exclusively at low gas loadings (Figures S8–S9) due to their low occupancy (1.30(18)–3.5(3)%). The disordered positions become invisible in the refined models at higher loadings than 2 mmol/g due to the poor density contrast against the background of the first layer sites. Therefore, no disordered propene molecules could be identified in the samples Zn-MOF-74-4, -6, -8, and -8_1K. Even though the population of the disordered sites cannot be entirely excluded at higher loadings than 2 mmol/g, the occupancy must be relatively low. The extremely short intermolecular distance of the disordered sites to the second adsorption layer does not allow the concurrent population of these two neighboring positions.

Regardless of the structural distortions in Zn-MOF-74-4, the evolution of lattice parameters as a function of gas loading reveals the overall unit cell expansion upon forming the second layer (Figure S15). Cell parameters are calculated solely from the peak positions, such that data on Zn-MOF-74-4 are also reliable. The channel elongation in the *c* direction from 6.8440(9) to 6.9049(9) Å emerging at 4 mmol/g loading is accompanied by a simultaneous channel contraction in diameter from 25.973(2) to 25.931(2) Å, which limits the swelling of the unit cell (Figure S14). The abrupt change in the cell parameters seems to be a consequence of the onset of the formation of the second adsorption layer. Further population of the second layer caused an increase of the cell volume in samples Zn-MOF-74-6 and -8 (Figure S15). The propene density within the channels of Zn-MOF-74 was calculated based on the number of propene molecules per unit cell and the experimental pore volume. These loadings are comparable to the dosed amounts (Figure S13). The refined total population of 22.6(4) propene molecules in a unit cell of Zn-MOF-74-8 yields a propene density of 0.707(14) g/cm^3^. Referencing this value to the liquid propene density at its boiling point (225.5 K, 102 kPa) of 0.609 g/cm^3^^54,55^ results in a relative density of 116(2)% at the same temperature (226 K).

All distances are reported on the data of Zn-MOF-74-8_1K (Figure 2), collected at 1 K for the accurate location of propene molecules within the pores. The orientation of the molecules remains comparable to the one in Zn-MOF-74-8. The first layer of propene coordinates via the C=C double bond (1.27(5) Å) to the OMS of Zn-MOF-74 (Figures 2c and S16). The Zn–C_1_ and Zn–C_2_ distances were 2.89(4) and 2.96(4) Å, respectively. These distances are longer than those reported for the adsorption of propene to Fe-MOF-74 (2.60(2)/2.56(2) Å)^17^ and Co-MOF-74 (2.66(5)/2.73(6) Å),^16^ while being comparable to the values of Mn-MOF-74 (3.03(9)/2.94(13) Å).^16^ The high promotional energy of Zn(II) (17.1 eV) is related to the poor π-back-donation.^56,57^ Zn alkene complexes are rare due to their instability. Several Zn alkenyl and Zn allyl compounds were reported to exhibit equivalent inter- or intramolecular Zn–C distances between 2.255(7) and 3.15(6) Å.^56,58,59^ Thus, the distances between Zn and C=C double bonds span a wide range, and the interaction of propene with Zn-MOF-74 stands in line with it. The distances between the C_2_ atoms of the first and the second layer ranged between 3.12(13) and 4.81(10) Å (Figure 2c and Table 1). A molecule of the second layer is situated between three propene molecules of the first layer. Two neighboring molecules on a shared Zn–O chain and a third molecule on the opposing side of the organic linker interact with a single second layer site (Figure 2c). The planes spanned by the three carbon atoms of P2 and the ones in P4 are approximately perpendicular to each other (84°). The first layer adsorbs in a side-on fashion, while the second displays an angle of 60° with respect to the organic linker.

**Table 1:** Intermolecular Distances between Propene Molecules of the First and the Second Adsorbate Layer, as Displayed in Figure 2c^a^

	C_2_–C_2_ dist. [Å]	shortest dist. [Å]
P_1_–P_4_	4.81(10)	2.86(14) (C_3_–C_1_)
P_2_–P_4_	4.22(10)	2.85(14) (C_1_–C_1_)
P_3_–P_4_	3.12(13)	2.68(12) (C_3_–C_2_)

a C_2_–C_2_ distances and the shortest distance between two carbon atoms of each pair of propene molecules, with the respective carbon atoms in brackets.

The observation of a second adsorbate layer and the differing density due to nanoconfinement agrees with GCMC calculations on the adsorbed propene within various MOF-74 derivatives.^51,52^ Co-MOF-74 was employed in the separation of equimolar propene/propane mixtures, where it exhibited increased selectivity toward propene over propane with increasing hydrocarbon pressures. This dependence contradicts general trends, where the selectivity toward one gas mixture component decreases with increasing gas pressures. The peculiar behavior of Co-MOF-74 was attributed to the dimensional match of the MOF channel diameter and the molecular size of propene such that the pore volume is efficiently filled and propane adsorption is suppressed.^51^ The explanation was supported by the expected selectivity decrease in ethene/ethane separation upon increasing gas pressures as a result of the mismatch between the pore diameter and the hydrocarbon size.^51^ Another study found that both ethane and ethene form a second adsorbate layer in Mg-MOF-74, while this is only possible for propene but not for propane. The different behavior of propene and propane, despite similar kinetic diameters, was attributed to the much higher dipole moment of propene compared to that of propane, allowing enhanced intermolecular interactions.^13,52^ In agreement with these two studies, we assume that the higher observed propene density results from the formation of a second adsorbate layer in close proximity to the first one. The former is enabled by sufficiently large intermolecular interactions,^52^ while the short intermolecular distances are caused by the restricted space within the channels (i.e., nanoconfinement) and the match between the sizes of the pore and double-layered propene (Figure 2b).^51^ The magnitude of the propene density increase is in agreement with the reported relative adsorbate density of 1-hexene in Zn-MOF-74. It was calculated by GCMC to be 14% higher than the liquid density, attributed to a more efficient packing of the olefin within the MOF channels than in the liquid phase.^35^

**Figure 3:**
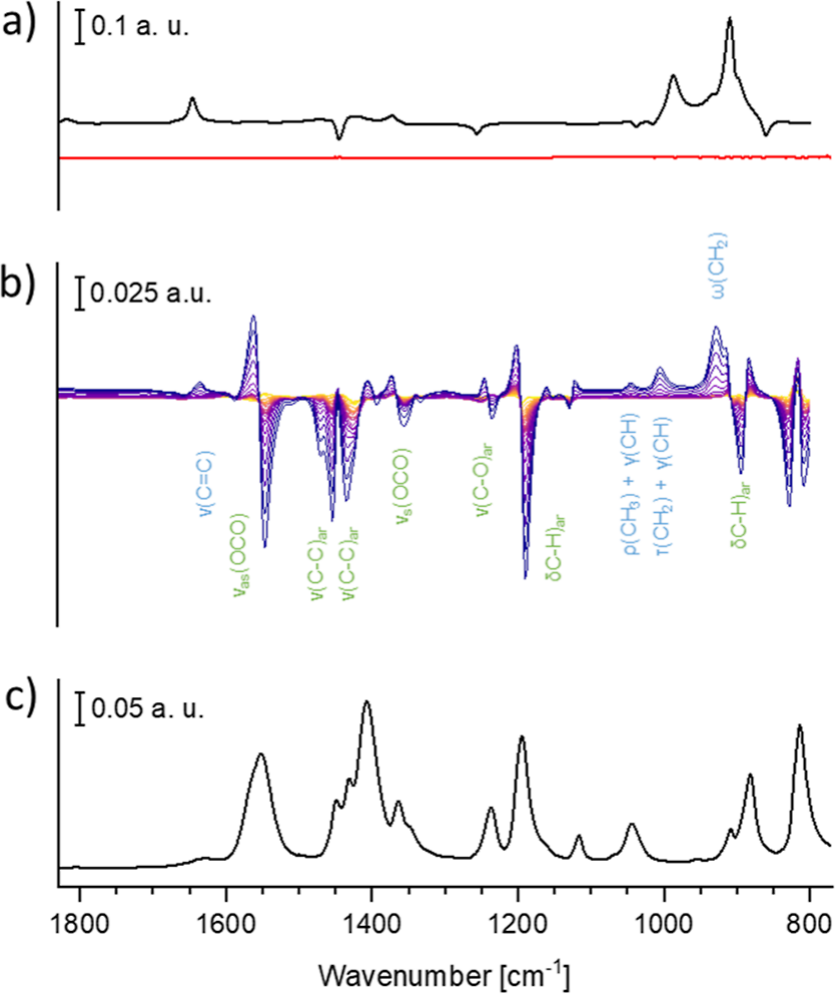
ATR-IR reference spectra of dissolved propene in cyclohexane (a, black) and gaseous propene (a, red) measured on a clean ZnSe crystal. Difference spectra acquired during propene gas adsorption on Zn-MOF-74 (b) and ATR-IR spectrum of Zn-MOF-74 (c). Green labels: MOF;^64,65^ light blue: propene^61,62^ (time domain: yellow to purple). Vibrational modes: ν_s_ and ν_as_, symmetric and asymmetric stretching; δ, bending; τ, twisting; ω, wagging; ρ, rocking.

We performed attenuated total reflection IR (ATR-IR) experiments to obtain a qualitative molecular perspective of the interaction between propene and Zn-MOF-74. No signals were detected when feeding gaseous propene (Figure 3a, red) on the clean ZnSe crystal, while propene dissolved in cyclohexane exhibited clear signals (Figure 3a, black). The peaks were shifted compared to the reference gas-phase spectrum, as a result of the condensed phase (Table 2). When a thin layer of Zn-MOF-74 on the ZnSe crystal was exposed to gas-phase propene in argon, signals similar to those observed in the spectrum of dissolved propene appeared. This shows that the MOF layer concentrates propene molecules within its porous structure and the space probed by IR radiation, similar to the change from gas phase to condensed phase. However, the further shift by 11 cm^–1^ in the position of the C=C stretch mode compared to dissolved propene accompanied by larger blue shifts of the two out-of-plane modes of the olefinic C–H vibration by 18 and 19 cm^–1^ confirms a more significant condensation effect induced by Zn-MOF-74 on the state of propene (Table 2). This suggests a stronger perturbation of the propene molecules upon interaction with the solid than the change of state from gaseous to condensed phase. Accordingly, the spectra obtained during the interaction of propene with the MOF display second-derivative-like profiles in correspondence of almost all vibrational modes of the MOF backbone, revealing a shift of the linker groups caused by the presence of the adsorbed olefin phase (Figure 3b, green labels). The interaction with propene also causes a loss of intensity in the signals of the perturbed groups, which can be explained considering that (i) the absorption coefficient of the linker signals involved decreases in the presence of propene or (ii) that the optical properties of the thin Zn-MOF-74 layer change in the presence of propene. Both observations are strong evidence of the interaction of propene with the MOF. These data demonstrate that all structural motifs of the 2,5-dioxidoterephthalate linker are involved in the physisorption of propene, in agreement with the NPD data, revealing all the organic entities being in proximity of less than 2.5 Å to at least one of the three positions of propene in the first adsorption layer. Even much longer distances have been reported to influence the vibrations of hydrogen bonds.^60^

**Table 2:** Experimental Vibrational Modes of Propene within Zn-MOF-74, Dissolved in Cyclohexane and in a Gas-Phase Reference Spectrum^a^

vibrational mode	Zn-MOF-74 [cm^–1^]	dissolved [cm^–1^]	ref. (g) [cm^–1^]^63^
ω(CH_2_)	927	909	912
τ(CH_2_) + γ(CH)	1006	987	990
ρ(CH_3_) + γ(CH)	1043		1044
ν(C=C)	1635	1646	1653
2ω(CH_2_)		1818	

a Band assignment according to refs (61) and (62).

The number of discrete adsorption sites and the activation energy of desorption were determined by TPD. The propene-loaded samples were heated with four different heating rates (β), ranging from 1 to 10 K/min. The curve with a heating rate of 10 K/min (Figure S18, red) exhibits three distinct peaks assigned to three unique adsorption sites. The peak with low intensity (at 270 K) corresponds to a site featuring little occupancy, probably the disordered one in the channel center in Figure S12. The second peak (at 302 K) and the main peak (at 333 K) were assigned to the second and first adsorption layers, respectively.

With the restraints described in the Supporting Information, varying β yields reasonably accurate *E*_A_des_ values.^66^ The analysis yielded *E*_A_des_ of propene on the OMS of Zn-MOF-74 as 42(5) kJ/mol. The value is slightly lower than the isosteric heat of adsorption reported previously (−47.6 kJ/mol).^16^ In the M-MOF-74 series, Zn-MOF-74 displayed the weakest interaction with propene.^16^ This weak interaction is in agreement with the long Zn–C distances obtained in the refinement of the NPD data.

The propene capacity of Zn-MOF-74 was probed with a physisorption experiment at 215 K to be 7.07 mmol/g at 0.95 *p*/*p*_0_, which is comparable to the number of adsorbed molecules, as determined by the NPD experiment (7.71(15) mmol/g). The total propene uptake according to NPD data and the physisorption experiment is comparable to the 6–7.8 mmol/g of propene adsorbed to Mg-MOF-74 at ambient temperature and 1 bar.^51,67^ The slight discrepancy between the values from the two experimental methods is rationalized by the lower BET surface area (870 m^2^/g) of the batch used in the physisorption experiment. However, both surface area values lie within the variability of the reported Zn-MOF-74 materials.^11,16,46,68−72^ The adsorption and desorption branches overlap (Figure S19). The formation of the first adsorption layer at the Zn OMS corresponds to roughly 5.8 mmol/g (6.0 mmol/g from NPD experiments), while the step at 0.95 *p*/*p*_0_ was attributed to propene molecules populating the second adsorption layer (1.3 mmol/g and 1.7 mmol/g from NPD). These data are thus in good agreement with the ones obtained under similar conditions by NPD at 226 K. Contrary to the most reported propene physisorption studies that were performed at room temperature,^16,17,51,52,73^ this experiment was conducted below the boiling point. We assume that the lower temperature is the reason why we observe the formation of the second adsorbate layer as a step at 0.95 *p*/*p*_0_, while it was not reported in earlier studies.

## Conclusions

In situ NPD is a suitable method to investigate the properties of nanoconfined adsorbate phases and to understand how they might deviate from the respective bulk properties. As a proof of concept, we studied the propene adsorption to Zn-MOF-74 by diffraction and corroborated the results by adsorption studies. The propene density within Zn-MOF-74 increases by 16(2)% compared to liquid propene. Sufficiently strong intermolecular interactions enable the formation of a second adsorbate layer, which, in combination with matching pore and olefin dimensions, leads to an efficient propene packing in the adsorbate phase. The second adsorbate layer forms close (3.12(13)–4.81(10) Å between C_2_ atoms) to the first one, as observed experimentally by in situ NPD. The distances of the Zn OMS to C_1_ and C_2_ were determined as 2.89(4) and 2.96(4) Å, respectively. All parts of the organic linker interact with propene and thereby contribute to its efficient packing, as shown by ATR-IR spectroscopy and corroborated by NPD. The formation of the second adsorbate layer was supported by TPD and propene physisorption experiments. The formation of the second layer was attributed to a step close to saturation in the physisorption experiment and to the shoulder displayed in the TPD peak. Finally, TPD experiments at various heating rates allowed the determination of *E*_A_des_ as 42(5) kJ/mol.

## Supporting Information Available

Characterization data of Zn-MOF-74; Rietveld refinements of additional samples; crystallographic data on all samples; structures of propene-loaded Zn-MOF-74; site occupancy; measured propene loadings; cell parameters; cell volumes as a function of the approximated propene loading; local structure of Zn-MOF-74-8_1K; difference Fourier maps of Zn-MOF-74-0, - 2, and -4; TPD experiments and the corresponding theories; and propene physisorption data (PDF)
Refined structure of Zn-MOF-74-0 (CIF)
Refined structure of Zn-MOF-74-1 (CIF)
Refined structure of Zn-MOF-74-2 (CIF)
Refined structure of Zn-MOF-74-4 (CIF)
Refined structure of Zn-MOF-74-6 (CIF)
Refined structure of Zn-MOF-74-8 (CIF)
Refined structure of Zn-MOF-74-8_1K (CIF)

## Supplementary Material

jp3c03225_si_001.pdf

jp3c03225_si_002.cif

jp3c03225_si_003.cif

jp3c03225_si_004.cif

jp3c03225_si_005.cif

jp3c03225_si_006.cif

jp3c03225_si_007.cif

jp3c03225_si_008.cif
