# Supplementary material for: In Situ Neutron Diffraction of Zn-MOF-74 Reveals Nanoconfinement-Induced Effects on Adsorbed Propene
Source: J Phys Chem C Nanomater Interfaces. 2023 Aug 16;127(33):16636–44. doi: 10.1021/acs.jpcc.3c03225 (PMC10461295; doi:10.1021/acs.jpcc.3c03225)
Supplement: Supplementary file 1 — jp3c03225_si_001.pdf [file jp3c03225_si_001.pdf]

Supporting Information

In Situ Neutron Diffraction of Zn-MOF-74 Reveals Nanoconfinement-Induced Effects on Adsorbed Propene

Patrick Gäumann[1], Davide Ferri[2], Denis Sheptyakov[3], Jeroen A. van Bokhoven[1,4], Przemyslaw Rzepka[1,4] and Marco Ranocchiari[1*]*

[1] Laboratory of Catalysis and Sustainable Chemistry, Paul Scherrer Institut, CH-5232 Villigen, Switzerland

[2] Bioenergy and Catalysis Laboratory, Paul Scherrer Institut, CH-5232 Villigen, Switzerland

[3] Laboratory for Neutron Scattering and Imaging, Paul Scherrer Institut, CH-5232 Villigen, Switzerland

[4] Institute of Chemical and Bioengineering, ETH Zurich, CH-8093 Zurich, Switzerland

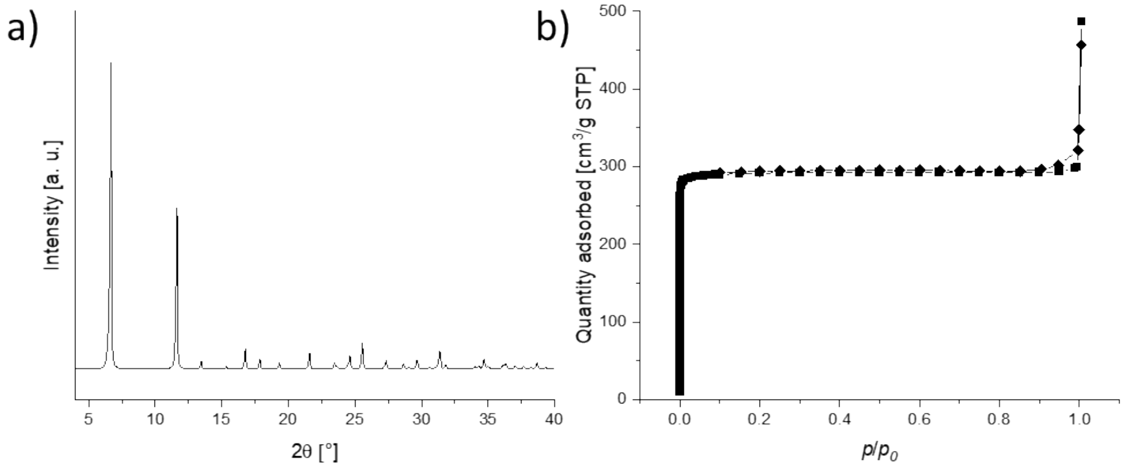

Figure S1: PXRD pattern (a) and nitrogen physisorption isotherm (b) of Zn-MOF-74. In the right graph, squares and diamonds correspond to data points of the adsorption and desorption branch, respectively.

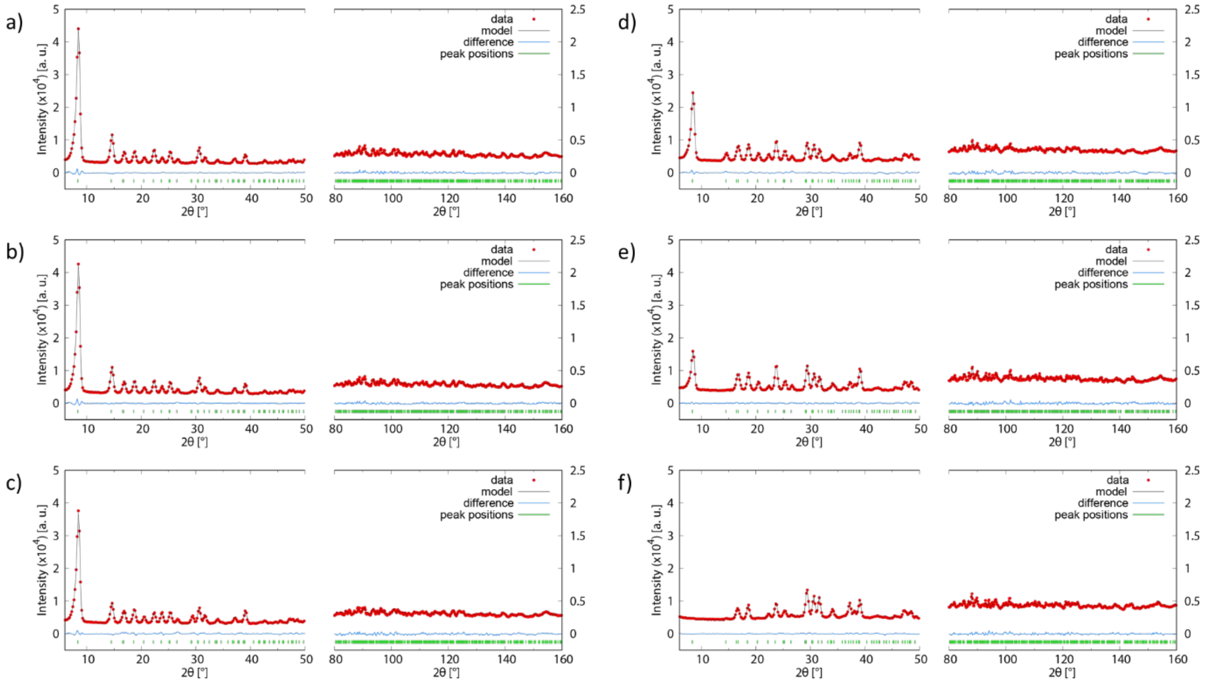

Figure S2: Rietveld refinements of Zn-MOF-74 charged with various amounts of deuterated propene (a: 0 mmol/g, b: 1 mmol/g, c: 2 mmol/g, d: 4 mmol/g, e: 6 mmol/g, f: 8 mmol/g). Data collected at 226 K.

Table S1: Crystallographic data of activated Zn-MOF-74-0, space group R-3, a = 25.9754(14), c = 6.8408(6), V = 3997.2(6), goodness of fit parameters, Rwp = 2.75735804, Rexp = 1.15762914, GoF = 2.38190104. Values in parentheses display the standard uncertainty of the refined value. Data collected at 226 K.

Atom	X	Y	Z	Occ.	B_{iso} [$Å^2$]	Multiplicity
C1	0.4218(5)	0.4049(6)	0.2604(18)	1	2.05(8)	18
C2	0.4602(5)	0.4542(6)	0.1206(19)	1	2.05(8)	18
C3	0.5555(6)	0.5465(6)	0.0781(17)	1	2.05(8)	18
C4	0.5155(6)	0.4978(6)	0.1966(16)	1	2.05(8)	18
H4	0.5260(9)	0.4994(10)	0.351(3)	1	2.0(4)	18
Zn1	0.6170(7)	0.6463(8)	0.363(2)	1	2.5(3)	18
O1	0.4405(7)	0.4078(6)	0.4393(19)	1	2.27(15)	18
O2	0.3720(6)	0.3652(7)	0.205(2)	1	2.27(15)	18
O3	0.6100(6)	0.5888(6)	0.160(2)	1	2.27(15)	18

Table S2: Crystallographic data of Zn-MOF-74-1, space group R-3, a = 25.9747(13), c = 6.8420(6), V = 3997.7(5), goodness of fit parameters, Rwp = 2.63770527, Rexp = 1.13349137, GoF = 2.3270625. Values in parentheses display the standard uncertainty of the refined value. Data collected at 226 K. Atoms with containing "f" and "p" in the label correspond to framework and propene, respectively. In the case of the propene atoms, numbers that are not bracketed depict the corresponding propene molecule, while bracketed numbers indicate the position within the molecule. E.g. Cp1(2) is carbon atom 2 in propene molecule 1.

Atom	X	Y	Z	Occ.	B_{iso} [Å^2]	Multiplicity
Cf1	0.4228(6)	0.4064(6)	0.260(2)	1	2.84(10)	18
Cf2	0.4630(6)	0.4567(7)	0.123(2)	1	2.84(10)	18
Cf3	0.5538(7)	0.5454(7)	0.077(2)	1	2.84(10)	18
Cf4	0.5147(7)	0.4979(7)	0.195(2)	1	2.84(10)	18
Hf	0.5271(11)	0.4970(13)	0.332(4)	1	3.0(5)	18
Znf1	0.6188(7)	0.6463(8)	0.360(2)	1	3.0(3)	18
Of1	0.4415(7)	0.4068(7)	0.437(2)	1	2.73(17)	18
Of2	0.3717(7)	0.3652(7)	0.204(3)	1	2.73(17)	18
Of3	0.6072(7)	0.5883(7)	0.163(2)	1	2.73(17)	18
Cp1(1)	0.40(3)	0.67(6)	0.04(9)	0.0130(18)	5(3)	18
Cp1(2)	0.387(18)	0.68(3)	0.22(9)	0.0130(18)	5(3)	18
Cp1(3)	0.325(17)	0.636(15)	0.29(5)	0.0130(18)	5(3)	18
Dp1(1)	0.45(3)	0.70(8)	0.99(14)	0.0130(18)	1.0(12)	18
Dp1(2)	0.37(4)	0.64(7)	0.94(6)	0.0130(18)	1.0(12)	18
Dp1(3)	0.42(3)	0.71(3)	0.32(14)	0.0130(18)	1.0(12)	18
Dp1(4)	0.30(3)	0.60(2)	0.17(7)	0.0130(18)	1.0(12)	18
Dp1(5)	0.33(2)	0.61(2)	0.42(7)	0.0130(18)	1.0(12)	18
Dp1(6)	0.30(3)	0.66(3)	0.33(7)	0.0130(18)	1.0(12)	18
Cp2(1)	0.691(5)	0.151(5)	0.828(11)	0.074(5)	5(3)	18
Cp2(2)	0.660(4)	0.157(4)	0.682(9)	0.074(5)	5(3)	18
Cp2(3)	0.690(3)	0.183(3)	0.492(9)	0.074(5)	5(3)	18
Dp2(1)	0.669(7)	0.131(7)	0.968(12)	0.074(5)	1.0(12)	18
Dp2(2)	0.739(6)	0.166(6)	0.807(14)	0.074(5)	1.0(12)	18
Dp2(3)	0.613(4)	0.142(6)	0.703(11)	0.074(5)	1.0(12)	18
Dp2(4)	0.737(3)	0.195(4)	0.499(10)	0.074(5)	1.0(12)	18
Dp2(5)	0.667(3)	0.150(3)	0.372(10)	0.074(5)	1.0(12)	18
Dp2(6)	0.687(4)	0.224(3)	0.462(12)	0.074(5)	1.0(12)	18

Table S3: Crystallographic data of Zn-MOF-74-2, space group R-3, a = 25.973(2), c = 6.8440(9), V = 3998.3(8), goodness of fit parameters, Rwp = 3.41087084, Rexp = 1.10388183, GoF = 3.08988766. Values in parentheses display the standard uncertainty of the refined value. Data collected at 226 K. Atoms with containing "f" and "p" in the label correspond to framework and propene, respectively. In the case of the propene atoms, numbers that are not bracketed depict the corresponding propene molecule, while bracketed numbers indicate the position within the molecule. E.g. Cp1(2) is carbon atom 2 in propene molecule 1.

Atom	X	Y	Z	Occ.	B_{iso} [Å^2]	Multiplicity
Cf1	0.4232(9)	0.4081(9)	0.263(3)	1	3.00(14)	18
Cf2	0.4658(9)	0.4591(10)	0.116(3)	1	3.00(14)	18
Cf3	0.5508(10)	0.5424(9)	0.073(3)	1	3.00(14)	18
Cf4	0.5128(10)	0.4970(10)	0.189(3)	1	3.00(14)	18
Hf	0.5326(14)	0.4971(17)	0.333(6)	1	3.0(7)	18
Znf1	0.6205(10)	0.6466(11)	0.357(3)	1	3.0(5)	18
Of1	0.4430(10)	0.4087(9)	0.439(3)	1	3.0(3)	18
Of2	0.3718(9)	0.3643(10)	0.209(4)	1	3.0(3)	18
Of3	0.6065(9)	0.5869(10)	0.165(4)	1	3.0(3)	18
Cp1(1)	0.40(2)	0.65(3)	0.03(10)	0.035(3)	5.0(16)	18
Cp1(2)	0.370(11)	0.647(19)	0.19(8)	0.035(3)	5.0(16)	18
Cp1(3)	0.307(9)	0.634(9)	0.18(4)	0.035(3)	5.0(16)	18
Dp1(1)	0.45(2)	0.66(4)	0.04(15)	0.035(3)	5.0(13)	18
Dp1(2)	0.38(3)	0.64(3)	0.89(8)	0.035(3)	5.0(13)	18
Dp1(3)	0.391(17)	0.65(3)	0.34(10)	0.035(3)	5.0(13)	18
Dp1(4)	0.293(17)	0.629(15)	0.02(5)	0.035(3)	5.0(13)	18
Dp1(5)	0.277(13)	0.592(11)	0.26(6)	0.035(3)	5.0(13)	18
Dp1(6)	0.303(18)	0.671(12)	0.25(5)	0.035(3)	5.0(13)	18
Cp2(1)	0.682(5)	0.146(5)	0.840(10)	0.188(6)	5.0(16)	18
Cp2(2)	0.650(3)	0.150(3)	0.695(7)	0.188(6)	5.0(16)	18
Cp2(3)	0.680(2)	0.183(2)	0.511(6)	0.188(6)	5.0(16)	18
Dp2(1)	0.660(7)	0.122(6)	0.975(11)	0.188(6)	5.0(13)	18
Dp2(2)	0.730(5)	0.167(6)	0.826(13)	0.188(6)	5.0(13)	18
Dp2(3)	0.601(3)	0.129(5)	0.709(10)	0.188(6)	5.0(13)	18
Dp2(4)	0.729(2)	0.201(3)	0.524(8)	0.188(6)	5.0(13)	18
Dp2(5)	0.662(3)	0.152(3)	0.385(7)	0.188(6)	5.0(13)	18
Dp2(6)	0.671(4)	0.220(3)	0.489(11)	0.188(6)	5.0(13)	18

Table S4: Crystallographic data of Zn-MOF-74-4, space group R-3, a = 25.931(2), c = 6.9049(11), V = 4020.9(9), goodness of fit parameters, Rwp = 3.55349703, Rexp = 1.04510728, GoF = 3.40012658. Values in parentheses display the standard uncertainty of the refined value. Data collected at 226 K. Atoms with containing "f" and "p" in the label correspond to framework and propene, respectively. In the case of the propene atoms, numbers that are not bracketed depict the corresponding propene molecule, while bracketed numbers indicate the position within the molecule. E.g. Cp1(2) is carbon atom 2 in propene molecule 1.

Atom	X	Y	Z	Occ.	B_{iso} [Å^2]	Multiplicity
Cf1	0.4241(11)	0.4083(12)	0.258(4)	1	2.84(19)	18
Cf2	0.4627(11)	0.4540(13)	0.114(4)	1	2.84(19)	18
Cf3	0.5485(11)	0.5433(11)	0.078(4)	1	2.84(19)	18
Cf4	0.5150(13)	0.4974(12)	0.197(3)	1	2.84(19)	18
Hf	0.5284(19)	0.496(2)	0.344(7)	1	2.9(9)	18
Znf1	0.6093(13)	0.6467(12)	0.376(4)	1	3.0(6)	18
Of1	0.4388(13)	0.4070(13)	0.441(4)	1	3.0(4)	18
Of2	0.3750(12)	0.3623(11)	0.186(4)	1	3.0(4)	18
Of3	0.6052(12)	0.5922(12)	0.166(4)	1	3.0(4)	18
Cp1(1)	0.290(3)	0.439(3)	0.204(17)	0.283(12)	5.0(7)	18
Cp1(2)	0.345(3)	0.483(3)	0.173(9)	0.283(12)	5.0(7)	18
Cp1(3)	0.3813(17)	0.5220(18)	0.338(6)	0.283(12)	5.0(7)	18
Dp1(1)	0.263(5)	0.410(5)	0.08(2)	0.283(12)	5.0(5)	18
Dp1(2)	0.270(2)	0.431(3)	0.35(2)	0.283(12)	5.0(5)	18
Dp1(3)	0.365(5)	0.491(4)	0.027(7)	0.283(12)	5.0(5)	18
Dp1(4)	0.354(3)	0.507(2)	0.474(7)	0.283(12)	5.0(5)	18
Dp1(5)	0.422(2)	0.519(3)	0.357(9)	0.283(12)	5.0(5)	18
Dp1(6)	0.394(2)	0.5691(19)	0.307(8)	0.283(12)	5.0(5)	18
Cp2(1)	0.609(3)	0.137(2)	0.709(6)	0.325(12)	5.0(7)	18
Cp2(2)	0.595(2)	0.139(2)	0.524(6)	0.325(12)	5.0(7)	18
Cp2(3)	0.6427(18)	0.1720(13)	0.378(5)	0.325(12)	5.0(7)	18
Dp2(1)	0.574(4)	0.113(3)	0.817(7)	0.325(12)	5.0(5)	18
Dp2(2)	0.656(4)	0.161(3)	0.754(6)	0.325(12)	5.0(5)	18
Dp2(3)	0.548(2)	0.115(3)	0.479(8)	0.325(12)	5.0(5)	18
Dp2(4)	0.6869(19)	0.193(2)	0.450(6)	0.325(12)	5.0(5)	18
Dp2(5)	0.640(2)	0.1407(16)	0.263(6)	0.325(12)	5.0(5)	18
Dp2(6)	0.636(3)	0.2075(18)	0.311(7)	0.325(12)	5.0(5)	18
Cp3(1)	0.201(2)	0.210(3)	0.540(5)	0.497(16)	5.0(7)	18
Cp3(2)	0.2011(12)	0.176(2)	0.394(4)	0.497(16)	5.0(7)	18
Cp3(3)	0.1606(9)	0.1636(11)	0.225(3)	0.497(16)	5.0(7)	18
Dp3(1)	0.231(2)	0.220(4)	0.665(5)	0.497(16)	5.0(5)	18
Dp3(2)	0.171(3)	0.229(2)	0.535(7)	0.497(16)	5.0(5)	18
Dp3(3)	0.231(2)	0.158(4)	0.400(5)	0.497(16)	5.0(5)	18
Dp3(4)	0.1338(18)	0.1859(19)	0.246(4)	0.497(16)	5.0(5)	18
Dp3(5)	0.1876(11)	0.1807(16)	0.090(4)	0.497(16)	5.0(5)	18
Dp3(6)	0.1302(17)	0.1147(12)	0.212(5)	0.497(16)	5.0(5)	18

Table S5: Crystallographic data of Zn-MOF-74-6, space group R-3, a = 25.9279(15), c = 6.9093(7), V = 4022.5(6), goodness of fit parameters, Rwp = 2.73434503, Rexp = 1.42730079, GoF = 1.91574548. Values in parentheses display the standard uncertainty of the refined value. Data collected at 226 K. Atoms with containing "f" and "p" in the label correspond to framework and propene, respectively. In the case of the propene atoms, numbers that are not bracketed depict the corresponding propene molecule, while bracketed numbers indicate the position within the molecule. E.g. Cp1(2) is carbon atom 2 in propene molecule 1.

Atom	X	Y	Z	Occ.	B_{iso} [Å^2]	Multiplicity
Cf1	0.4207(8)	0.4034(9)	0.248(3)	1	2.57(13)	18
Cf2	0.4642(9)	0.4529(10)	0.120(2)	1	2.57(13)	18
Cf3	0.5561(9)	0.5478(8)	0.086(3)	1	2.57(13)	18
Cf4	0.5154(9)	0.4994(8)	0.193(2)	1	2.57(13)	18
Hf	0.5204(15)	0.4944(17)	0.342(5)	1	3.0(6)	18
Znf1	0.6156(10)	0.6491(11)	0.358(3)	1	3.0(4)	18
Of1	0.4354(11)	0.4058(11)	0.438(3)	1	3.0(2)	18
Of2	0.3741(10)	0.3622(10)	0.188(3)	1	3.0(2)	18
Of3	0.6092(10)	0.5935(10)	0.165(3)	1	3.0(2)	18
Cp1(1)	0.307(7)	0.478(8)	0.111(19)	0.134(12)	5.0(6)	18
Cp1(2)	0.363(6)	0.511(5)	0.171(10)	0.134(12)	5.0(6)	18
Cp1(3)	0.377(3)	0.518(4)	0.383(9)	0.134(12)	5.0(6)	18
Dp1(1)	0.297(10)	0.472(10)	6.95(2)	0.134(12)	5.0(5)	18
Dp1(2)	0.270(5)	0.457(8)	0.22(3)	0.134(12)	5.0(5)	18
Dp1(3)	0.399(8)	0.532(7)	0.065(11)	0.134(12)	5.0(5)	18
Dp1(4)	0.334(4)	0.494(5)	0.467(14)	0.134(12)	5.0(5)	18
Dp1(5)	0.406(5)	0.500(5)	0.420(12)	0.134(12)	5.0(5)	18
Dp1(6)	0.399(5)	0.567(4)	0.420(13)	0.134(12)	5.0(5)	18
Cp2(1)	0.667(4)	0.144(4)	0.731(8)	0.266(12)	5.0(6)	18
Cp2(2)	0.631(3)	0.150(3)	0.606(5)	0.266(12)	5.0(6)	18
Cp2(3)	0.653(2)	0.1770(16)	0.411(5)	0.266(12)	5.0(6)	18
Dp2(1)	0.650(5)	0.124(5)	0.874(9)	0.266(12)	5.0(5)	18
Dp2(2)	0.714(4)	0.160(4)	0.692(10)	0.266(12)	5.0(5)	18
Dp2(3)	0.584(3)	0.134(4)	0.646(7)	0.266(12)	5.0(5)	18
Dp2(4)	0.701(2)	0.190(2)	0.398(6)	0.266(12)	5.0(5)	18
Dp2(5)	0.627(2)	0.1441(19)	0.296(6)	0.266(12)	5.0(5)	18
Dp2(6)	0.649(3)	0.217(2)	0.395(8)	0.266(12)	5.0(5)	18
Cp3(1)	0.580(10)	0.366(13)	0.09(3)	0.074(4)	5.0(6)	18
Cp3(2)	0.634(9)	0.375(10)	0.044(19)	0.074(4)	5.0(6)	18
Cp3(3)	0.647(7)	0.365(6)	0.841(17)	0.074(4)	5.0(6)	18
Dp3(1)	0.570(13)	0.373(17)	0.24(4)	0.074(4)	5.0(5)	18
Dp3(2)	0.545(9)	0.351(13)	0.98(4)	0.074(4)	5.0(5)	18
Dp3(3)	0.669(11)	0.390(13)	0.155(18)	0.074(4)	5.0(5)	18
Dp3(4)	0.606(8)	0.350(8)	0.75(2)	0.074(4)	5.0(5)	18
Dp3(5)	0.684(8)	0.407(7)	0.78(2)	0.074(4)	5.0(5)	18
Dp3(6)	0.660(9)	0.330(7)	0.84(3)	0.074(4)	5.0(5)	18
Cp4(1)	0.2015(15)	0.203(2)	0.530(4)	0.525(16)	5.0(6)	18
Cp4(2)	0.1991(10)	0.1690(19)	0.381(3)	0.525(16)	5.0(6)	18
Cp4(3)	0.1560(8)	0.1569(10)	0.219(3)	0.525(16)	5.0(6)	18
Dp4(1)	0.2332(17)	0.212(3)	0.649(4)	0.525(16)	5.0(5)	18

Dp4(2)	0.172(2)	0.222(2)	0.535(5)	0.525(16)	5.0(5)	18
Dp4(3)	0.2287(16)	0.150(3)	0.376(4)	0.525(16)	5.0(5)	18
Dp4(4)	0.1301(14)	0.1797(15)	0.249(3)	0.525(16)	5.0(5)	18
Dp4(5)	0.1809(10)	0.1740(12)	0.081(3)	0.525(16)	5.0(5)	18
Dp4(6)	0.1250(13)	0.1081(10)	0.207(4)	0.525(16)	5.0(5)	18

Table S6: Crystallographic data of Zn-MOF-74-8, space group R-3, a = 25.9610(13), c = 6.9204(6), V = 4039.3(5), goodness of fit parameters, Rwp = 2.14753074, Rexp = 0.947959757, GoF = 2.26542396. Values in parentheses display the standard uncertainty of the refined value. Data collected at 226 K. Atoms with containing "f" and "p" in the label correspond to framework and propene, respectively. In the case of the propene atoms, numbers that are not bracketed depict the corresponding propene molecule, while bracketed numbers indicate the position within the molecule. E.g. Cp1(2) is carbon atom 2 in propene molecule 1.

Atom	X	Y	Z	Occ.	B_{iso} [Å^2]	Multiplicity
Cf1	0.4215(7)	0.4039(8)	0.250(2)	1	1.96(11)	18
Cf2	0.4609(7)	0.4532(7)	0.124(2)	1	1.96(11)	18
Cf3	0.5555(8)	0.5463(7)	0.076(2)	1	1.96(11)	18
Cf4	0.5176(7)	0.4988(7)	0.200(2)	1	1.96(11)	18
Hf	0.5230(11)	0.4996(12)	0.348(4)	1	1.6(5)	18
Znf1	0.6159(9)	0.6485(10)	0.374(3)	1	3.0(4)	18
Of1	0.4356(9)	0.4081(10)	0.431(2)	1	2.3(2)	18
Of2	0.3747(8)	0.3621(8)	0.194(3)	1	2.3(2)	18
Of3	0.6056(8)	0.5884(8)	0.163(3)	1	2.3(2)	18
Cp1(1)	0.308(3)	0.472(2)	0.097(5)	0.351(12)	5.0(5)	18
Cp1(2)	0.359(2)	0.4996(17)	0.195(3)	0.351(12)	5.0(5)	18
Cp1(3)	0.3587(14)	0.5025(12)	0.411(3)	0.351(12)	5.0(5)	18
Dp1(1)	0.309(4)	0.470(3)	6.938(5)	0.351(12)	5.0(4)	18
Dp1(2)	0.266(2)	0.452(3)	0.175(8)	0.351(12)	5.0(4)	18
Dp1(3)	0.402(3)	0.520(2)	0.117(5)	0.351(12)	5.0(4)	18
Dp1(4)	0.3119(14)	0.4794(17)	0.463(5)	0.351(12)	5.0(4)	18
Dp1(5)	0.3823(18)	0.4803(17)	0.473(4)	0.351(12)	5.0(4)	18
Dp1(6)	0.3814(18)	0.5498(13)	0.458(4)	0.351(12)	5.0(4)	18
Cp2(1)	0.662(3)	0.144(3)	0.751(6)	0.322(12)	5.0(5)	18
Cp2(2)	0.625(2)	0.1460(18)	0.620(4)	0.322(12)	5.0(5)	18
Cp2(3)	0.6484(14)	0.1725(13)	0.425(4)	0.322(12)	5.0(5)	18
Dp2(1)	0.646(4)	0.125(3)	0.894(6)	0.322(12)	5.0(4)	18
Dp2(2)	0.710(3)	0.162(3)	0.715(7)	0.322(12)	5.0(4)	18
Dp2(3)	0.578(2)	0.128(3)	0.656(5)	0.322(12)	5.0(4)	18
Dp2(4)	0.6968(15)	0.1881(19)	0.416(5)	0.322(12)	5.0(4)	18
Dp2(5)	0.6243(17)	0.1383(15)	0.312(4)	0.322(12)	5.0(4)	18
Dp2(6)	0.641(2)	0.2108(16)	0.402(6)	0.322(12)	5.0(4)	18
Cp3(1)	0.5746(19)	0.375(3)	0.192(6)	0.280(5)	5.0(5)	18
Cp3(2)	0.6216(17)	0.368(2)	0.165(3)	0.280(5)	5.0(5)	18
Cp3(3)	0.6397(13)	0.3625(14)	0.964(3)	0.280(5)	5.0(5)	18
Dp3(1)	0.561(3)	0.380(4)	0.340(7)	0.280(5)	5.0(4)	18
Dp3(2)	0.5492(18)	0.377(3)	0.067(7)	0.280(5)	5.0(4)	18
Dp3(3)	0.647(2)	0.367(3)	0.290(3)	0.280(5)	5.0(4)	18

Dp3(4)	0.6090(14)	0.3652(18)	0.858(4)	0.280(5)	5.0(4)	18
Dp3(5)	0.6859(13)	0.3991(16)	0.937(5)	0.280(5)	5.0(4)	18
Dp3(6)	0.6378(16)	0.3189(15)	0.949(5)	0.280(5)	5.0(4)	18
Cp4(1)	0.196(3)	0.207(4)	0.546(6)	0.297(15)	5.0(5)	18
Cp4(2)	0.2011(16)	0.177(3)	0.396(5)	0.297(15)	5.0(5)	18
Cp4(3)	0.1609(13)	0.1629(14)	0.225(4)	0.297(15)	5.0(5)	18
Dp4(1)	0.226(3)	0.217(5)	0.672(7)	0.297(15)	5.0(4)	18
Dp4(2)	0.163(4)	0.221(3)	0.544(8)	0.297(15)	5.0(4)	18
Dp4(3)	0.234(2)	0.163(5)	0.398(6)	0.297(15)	5.0(4)	18
Dp4(4)	0.130(2)	0.181(2)	0.250(6)	0.297(15)	5.0(4)	18
Dp4(5)	0.1882(15)	0.1838(19)	0.094(5)	0.297(15)	5.0(4)	18
Dp4(6)	0.134(2)	0.1139(14)	0.205(7)	0.297(15)	5.0(4)	18

Table S7: Crystallographic data of Zn-MOF-74-8_1K, space group R-3, a = 25.8956(15), c = 6.9200(6), V = 4018.7(6), goodness of fit parameters, Rwp = 2.8709897, Rexp = 0.834896507, GoF = 3.43873723. Values in parentheses display the standard uncertainty of the refined value. Data collected at 1 K. Atoms with containing "f" and "p" in the label correspond to framework and propene, respectively. In the case of the propene atoms, numbers that are not bracketed depict the corresponding propene molecule, while bracketed numbers indicate the position within the molecule. E.g. Cp1(2) is carbon atom 2 in propene molecule 1.

Atom	X	Y	Z	Occ.	B_{iso} [Å^2]	Multiplicity
Cf1	0.4219(7)	0.4055(8)	0.253(2)	1	1.52(11)	18
Cf2	0.4594(8)	0.4502(9)	0.122(2)	1	1.52(11)	18
Cf3	0.5528(8)	0.5444(8)	0.072(2)	1	1.52(11)	18
Cf4	0.5131(8)	0.4971(8)	0.191(2)	1	1.52(11)	18
Hf	0.5235(18)	0.500(2)	0.329(6)	1	4.1(8)	18
Znf	0.6153(9)	0.6423(11)	0.356(3)	1	1.8(4)	18
Of1	0.4374(9)	0.4076(9)	0.437(3)	1	1.8(2)	18
Of2	0.3720(9)	0.3607(9)	0.184(3)	1	1.8(2)	18
Of3	0.6134(9)	0.5947(9)	0.170(3)	1	1.8(2)	18
Cp1(1)	0.2002(13)	0.1969(12)	0.516(4)	0.880(11)	3.7(3)	18
Cp1(2)	0.1923(12)	0.1686(13)	0.359(4)	0.880(11)	3.7(3)	18
Cp1(3)	0.1548(13)	0.1603(14)	0.180(4)	0.880(11)	3.7(3)	18
Dp1(1)	0.2244(13)	0.2017(14)	0.642(5)	0.880(11)	5.0(3)	18
Dp1(2)	0.1722(14)	0.2156(13)	0.529(4)	0.880(11)	5.0(3)	18
Dp1(3)	0.2182(14)	0.1483(14)	0.370(4)	0.880(11)	5.0(3)	18
Dp1(4)	0.1250(13)	0.1799(15)	0.185(5)	0.880(11)	5.0(3)	18
Dp1(5)	0.1873(15)	0.1685(13)	0.064(4)	0.880(11)	5.0(3)	18
Dp1(6)	0.1212(13)	0.1107(15)	0.160(5)	0.880(11)	5.0(3)	18
Cp2(1)	0.385(6)	0.557(6)	0.47(2)	0.162(5)	3.7(3)	18
Cp2(2)	0.375(5)	0.603(5)	0.470(11)	0.162(5)	3.7(3)	18
Cp2(3)	0.368(3)	0.628(3)	0.283(8)	0.162(5)	3.7(3)	18
Dp2(1)	0.389(8)	0.539(8)	0.61(3)	0.162(5)	5.0(3)	18
Dp2(2)	0.388(7)	0.538(5)	0.33(3)	0.162(5)	5.0(3)	18
Dp2(3)	0.371(5)	0.623(6)	0.608(8)	0.162(5)	5.0(3)	18
Dp2(4)	0.373(4)	0.603(3)	0.161(11)	0.162(5)	5.0(3)	18
Dp2(5)	0.324(4)	0.625(4)	0.276(11)	0.162(5)	5.0(3)	18
Dp2(6)	0.404(4)	0.676(3)	0.272(11)	0.162(5)	5.0(3)	18

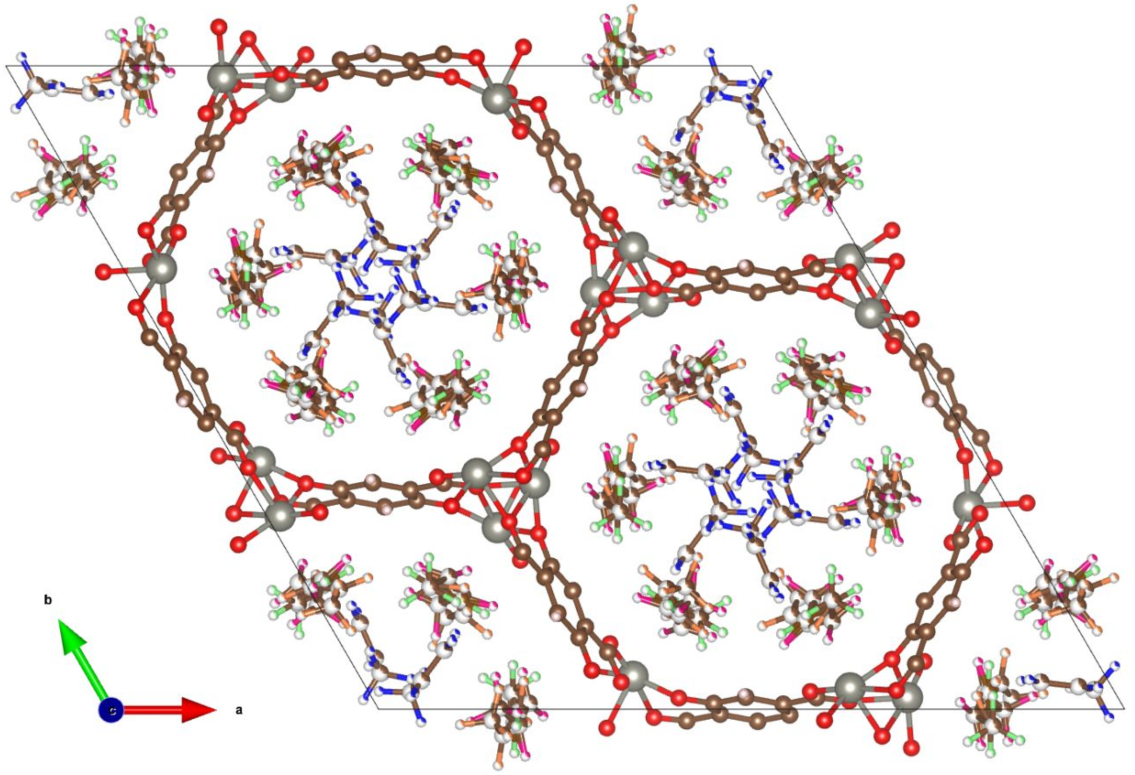

Figure S3: Four different propene positions in Zn-MOF-74-8. Data collected at 226 K, space group R-3, a = 25.9610(13), c = 6.9204(6), V = 4039.3(5), goodness of fit parameters, Rwp = 2.14753074, Rexp = 0.947959757, GoF = 2.26542396. The colored fraction of the propene atoms corresponds to the occupancy of that site. Color code D in propene molecules: light green, position 1; orange, position 2; pink, position 3; blue, second layer. Color code atoms: brown, C; white, H; red, O; grey, Zn.

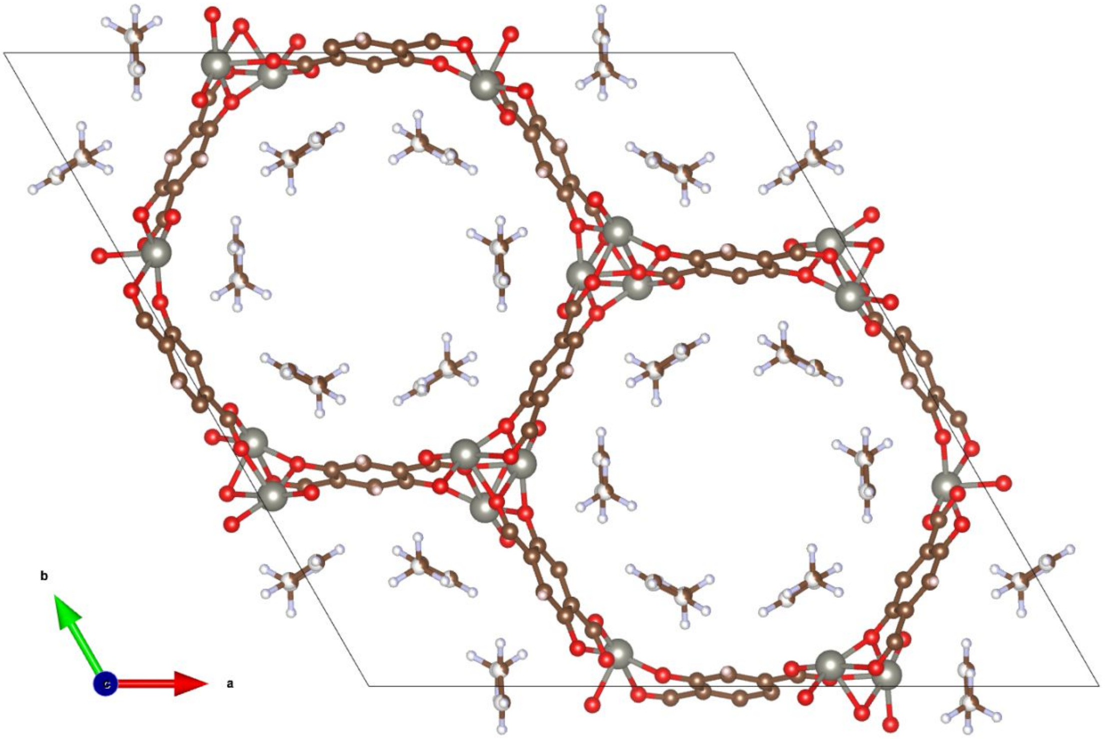

Figure S4: Representation of the first propene adsorption layer in Zn-MOF-74-8, position 1. Data collected at 226 K, space group R-3, a = 25.9610(13), c = 6.9204(6), V = 4039.3(5), goodness of fit parameters, Rwp = 2.14753074,

Rexp = 0.947959757, GoF = 2.26542396. The colored fraction of the propene carbon atoms corresponds to the occupancy of that site. Color code: brown, C; white, H; red, O; grey, Zn.

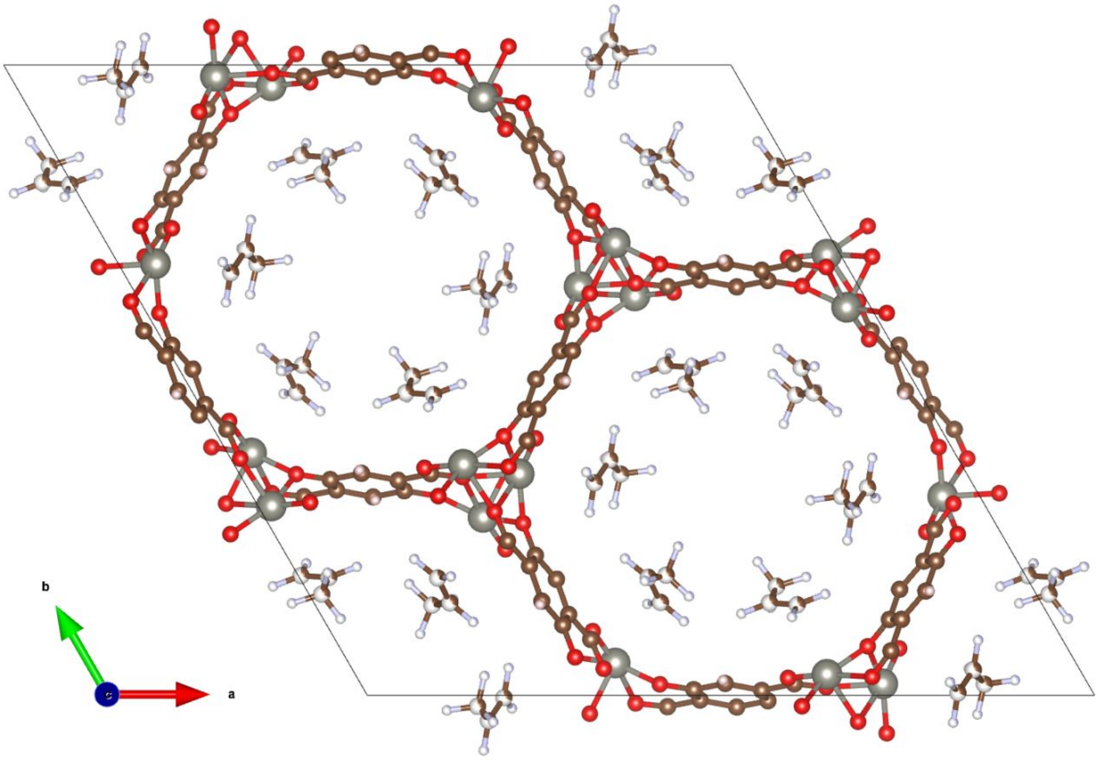

Figure S5: Representation of the first propene adsorption layer in Zn-MOF-74-8, position 2. Data collected at 226 K, space group R-3, a = 25.9610(13), c = 6.9204(6), V = 4039.3(5), goodness of fit parameters, Rwp = 2.14753074,
Rexp = 0.947959757, GoF = 2.26542396. The colored fraction of the propene carbon atoms corresponds to the occupancy of that site. Color code: brown, C; white, H; red, O; grey, Zn.

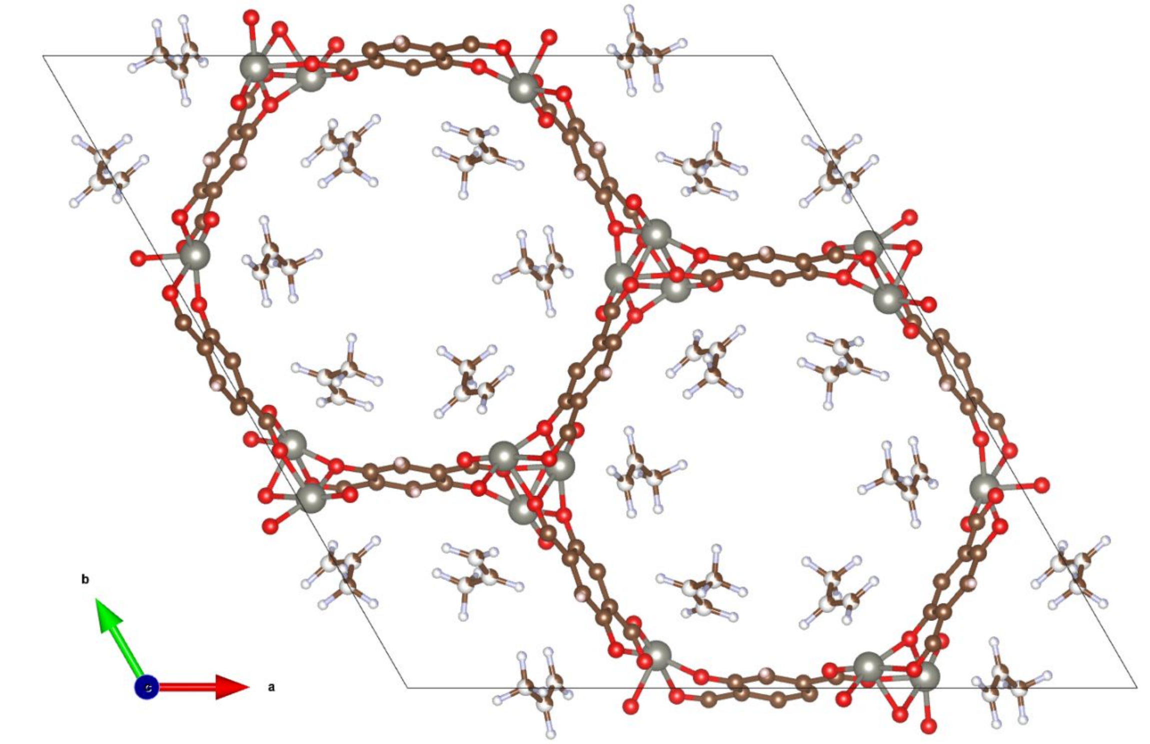

Figure S6: Representation of the first propene adsorption layer in Zn-MOF-74-8, position 3. Data collected at 226 K, space group R-3, a = 25.9610(13), c = 6.9204(6), V = 4039.3(5), goodness of fit parameters, Rwp = 2.14753074, Rexp = 0.947959757, GoF = 2.26542396. The colored fraction of the propene carbon atoms corresponds to the occupancy of that site. Color code: brown, C; white, H; red, O; grey, Zn.

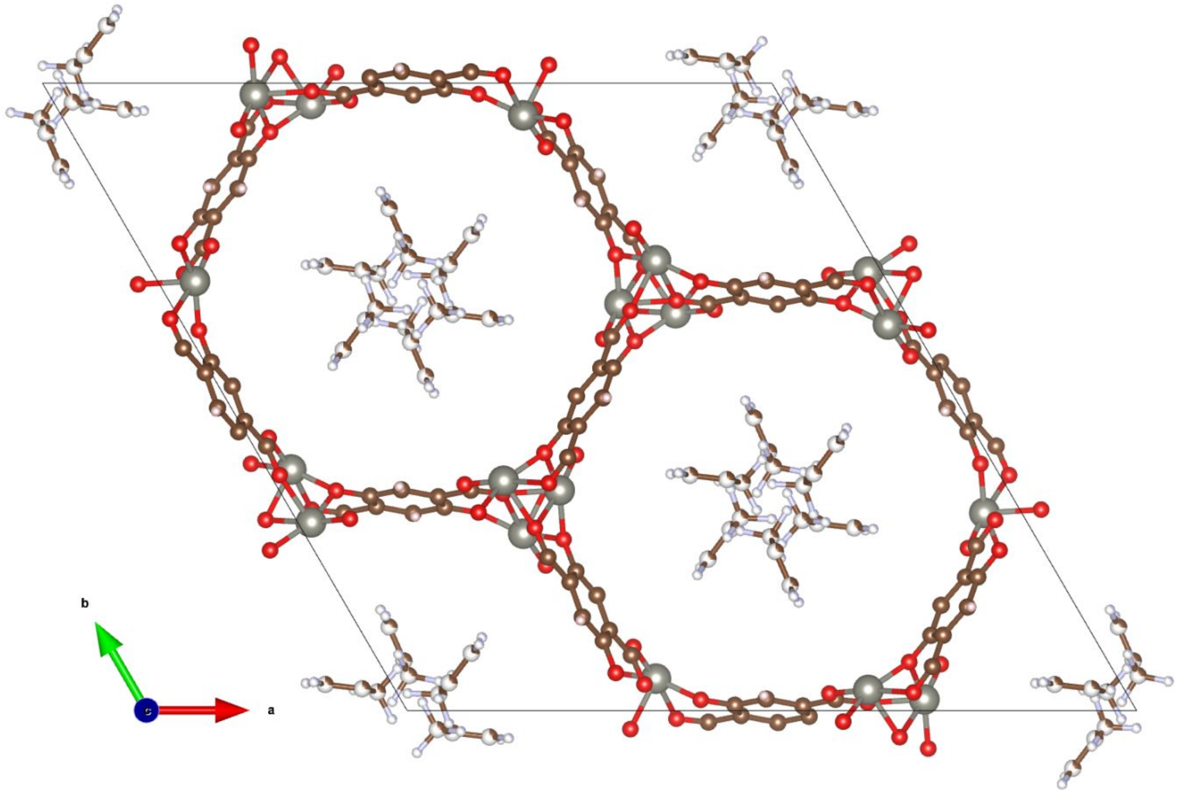

Figure S7: Representation of the second propene adsorption layer in Zn-MOF-74-8. Data collected at 226 K, space group R-3, a = 25.9610(13), c = 6.9204(6), V = 4039.3(5), goodness of fit parameters, Rwp = 2.14753074, Rexp = 0.947959757,

GoF = 2.26542396. The colored fraction of the propene carbon atoms corresponds to the occupancy of that site. Color code: brown, C; white, H; red, O; grey, Zn.

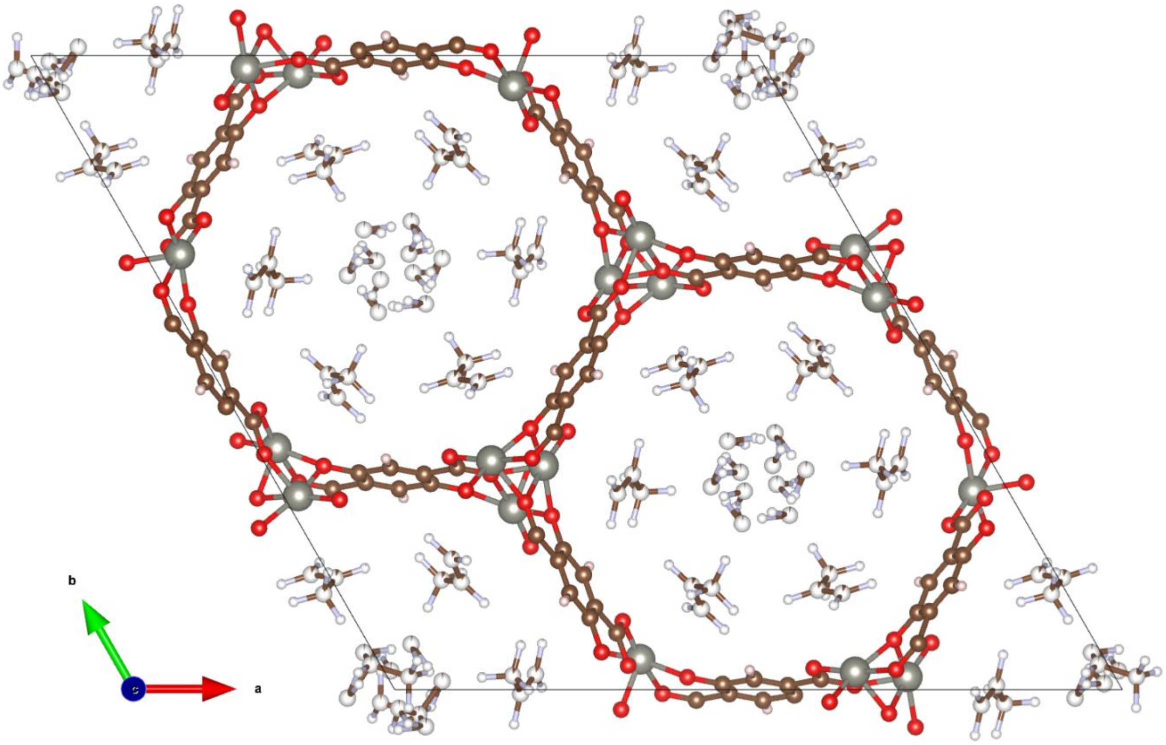

Figure S8: Structure of deuterated propene in Zn-MOF-74-1. Data collected at 226 K, space group R-3, a = 25.9747(13), c = 6.8420(6), V = 3997.7(5), goodness of fit parameters, Rwp = 2.63770527, Rexp = 1.13349137, GoF = 2.3270625. The colored fraction of the propene carbon atoms corresponds to the occupancy of that site. Color code: brown, C; white, H; red, O; grey, Zn.

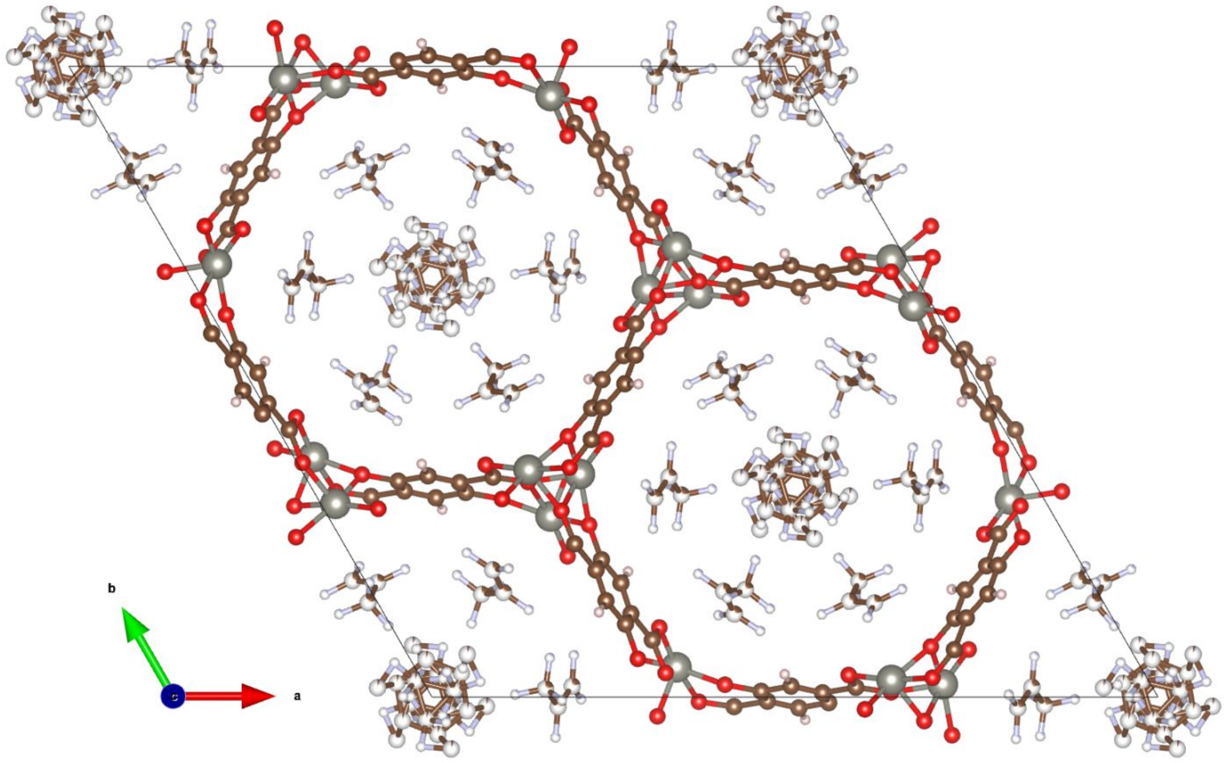

Figure S9: Structure of deuterated propene in Zn-MOF-74-2. Data collected at 226 K, space group R-3, a = 25.973(2), c = 6.8440(9), V = 3998.3(8), goodness of fit parameters, Rwp = 3.41087084, Rexp = 1.10388183, GoF = 3.08988766. The colored fraction of the propene carbon atoms corresponds to the occupancy of that site. Color code: brown, C; white, H; red, O; grey, Zn.

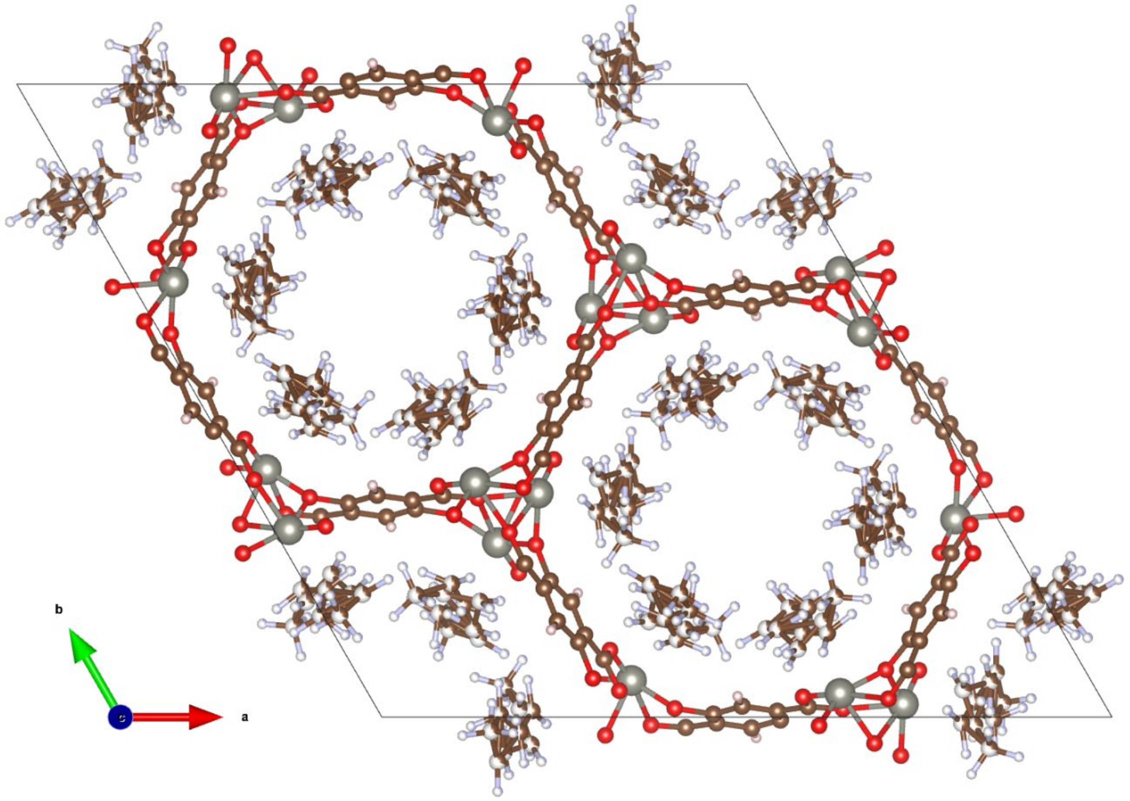

Figure S10: Structure of deuterated propene in Zn-MOF-74-4. Data collected at 226 K, space group R-3, a = 25.931(2), c = 6.9049(11), V = 4020.9(9), goodness of fit parameters, Rwp = 3.55349703, Rexp = 1.04510728, GoF = 3.40012658. The colored fraction of the propene carbon atoms corresponds to the occupancy of that site. Color code: brown, C; white, H; red, O; grey, Zn.

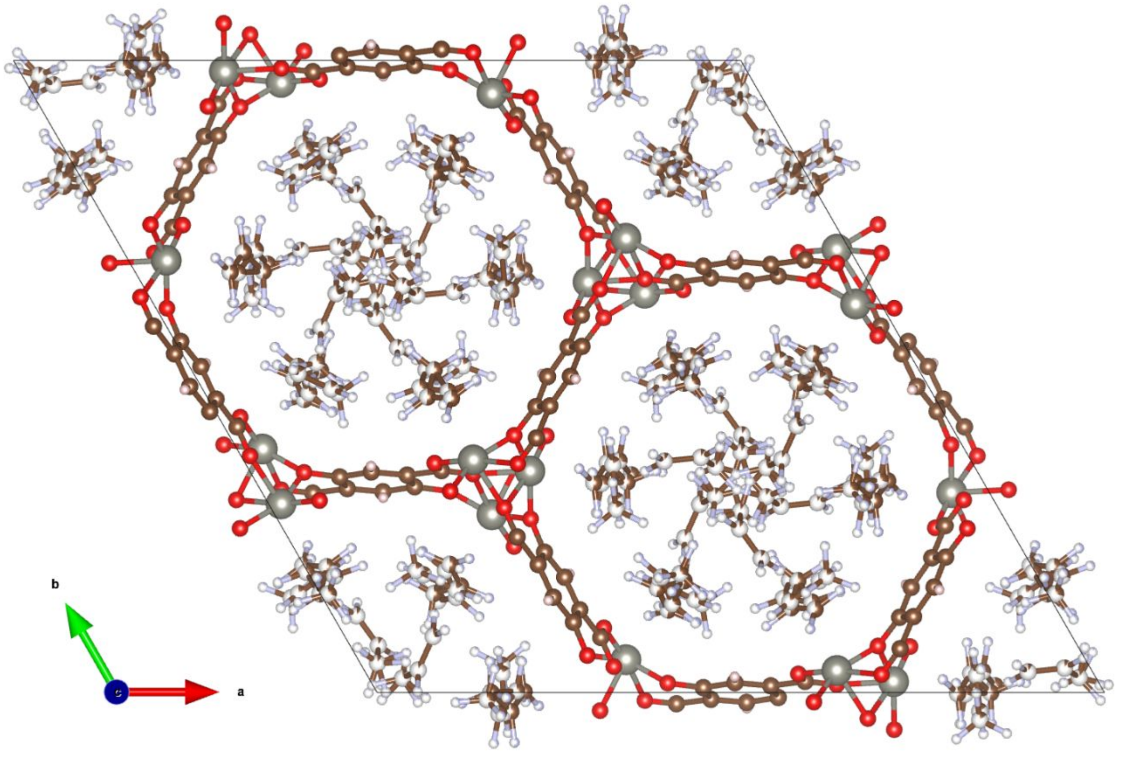

Figure S11: Structure of deuterated propene in Zn-MOF-74-6. Data collected at 226 K, space group R-3, a = 25.9279(15), c = 6.9093(7), V = 4022.5(6), goodness of fit parameters, Rwp = 2.73434503, Rexp = 1.42730079, GoF = 1.91574548. The

colored fraction of the propene carbon atoms corresponds to the occupancy of that site. Color code: brown, C; white, H; red, O; grey, Zn.

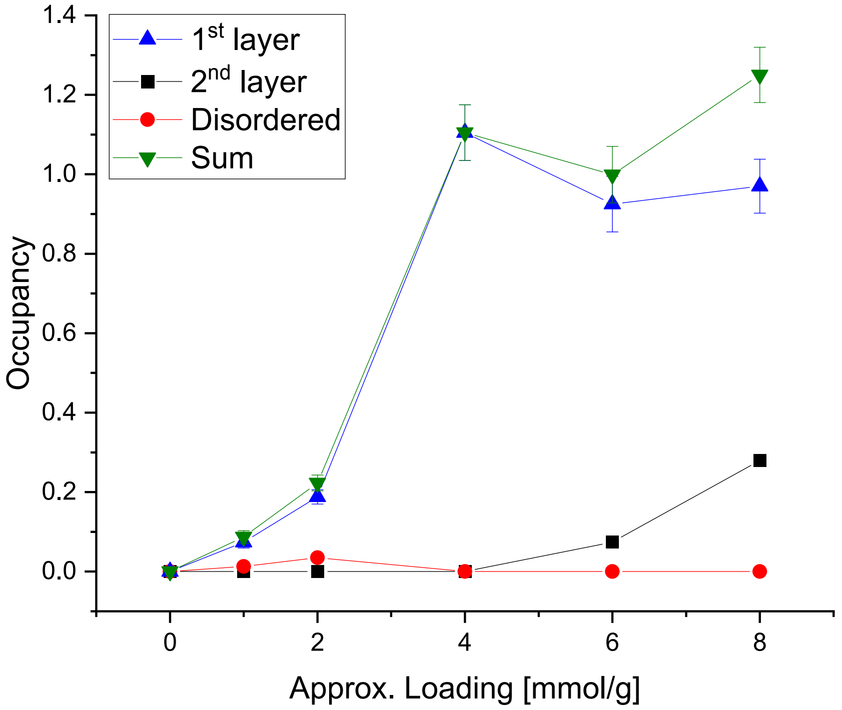

Figure S12: Occupancy of various sites as a function of the dosed amount of deuterated propene. Sites 1 to 3 are combined in the first adsorption layer. The error bars reflect three standard uncertainties of the refined occupancy. Lines are guides to the eye. Data collected at 226 K.

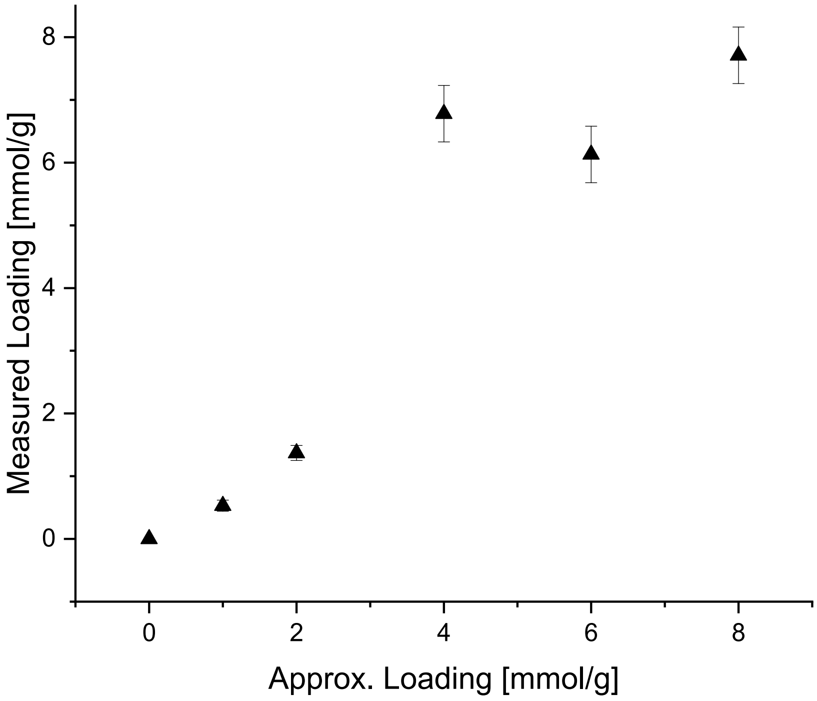

Figure S13: Measured loadings as refined against the approximated loadings dosed. The error bars reflect three standard uncertainties of the measured loading as refined from the NPD data. Data collected at 226 K.

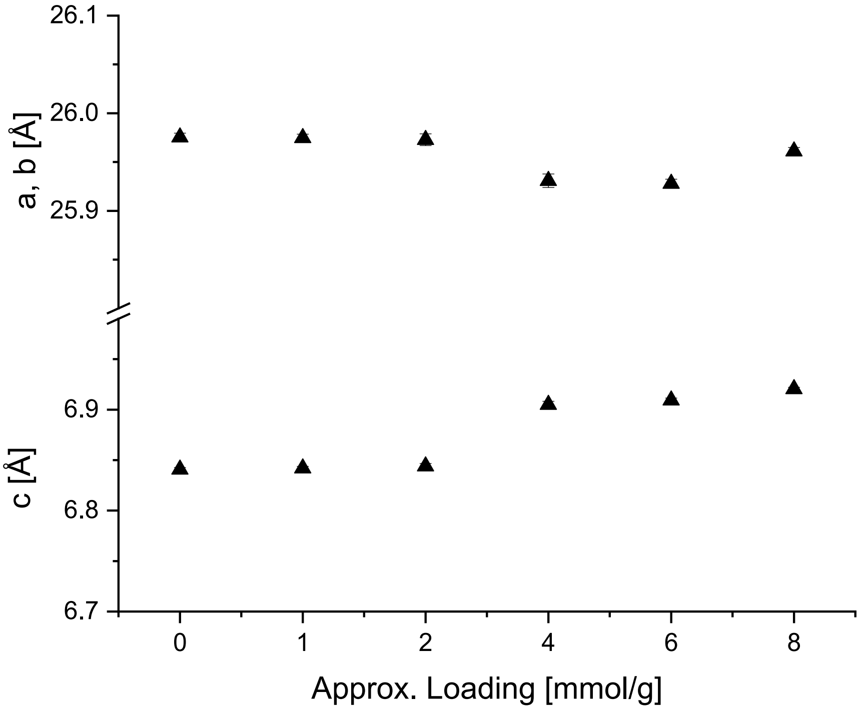

Figure S14: Cell parameters as a function of the approximated loadings. The error bars reflect three standard uncertainties of the refined cell parameters. Data collected at 226 K.

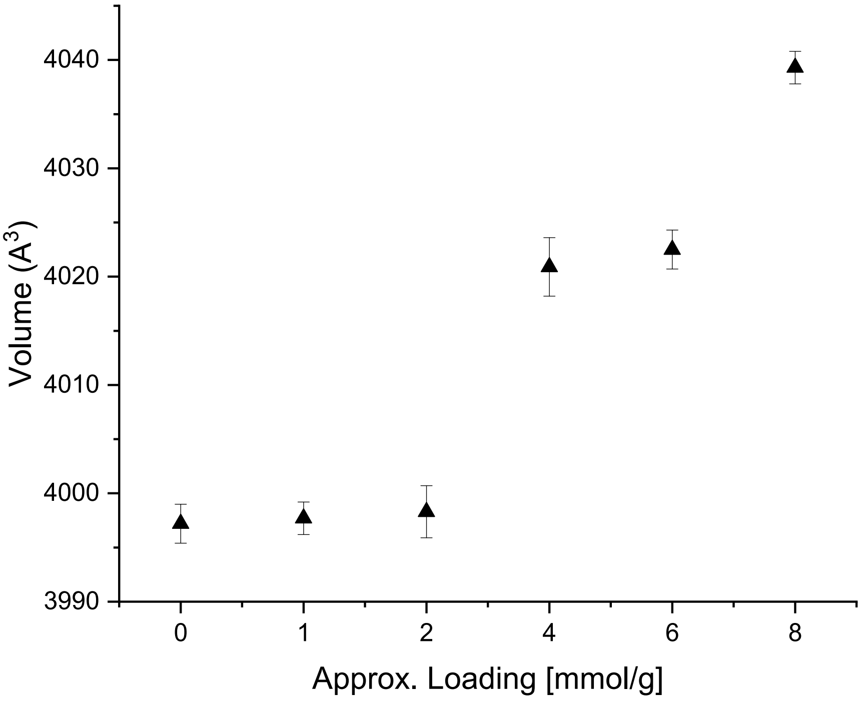

Figure S15: Cell volumes as a function of the approximated loadings. The error bars reflect three standard uncertainties of the refined volumes. Data collected at 226 K.

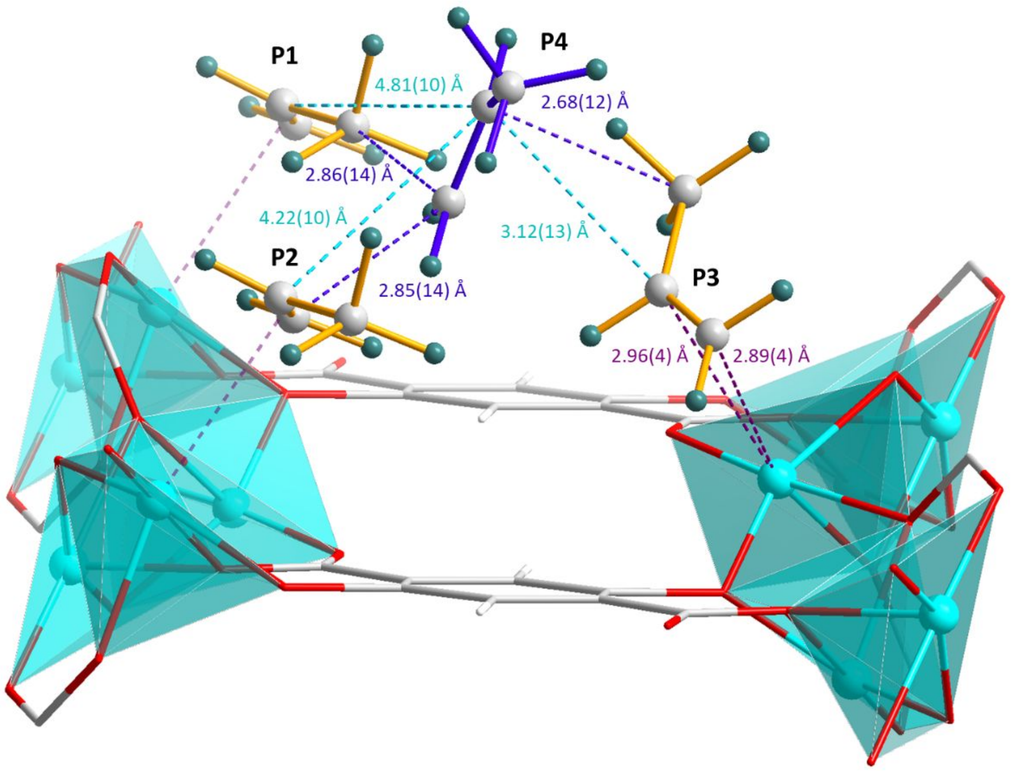

Figure S16: Local structure in a fraction of the pore of Zn-MOF-74-8_1K. Propene molecules of the first adsorption layer (P1, P2, P3) are drawn with yellow bonds, while second-layer propene molecules (P4) feature dark blue ones. The adsorption of propene molecules to Zn(II) sites (cyan spheres) is depicted by purple lines, while the distances Zn-C_1 and Zn-C_2 are represented in a darker shade of purple. The C_2-C_2 distance between a molecule of the first and one of the second layer is displayed in cyan, whereas the shortest distance is shown in dark blue. Color code: grey, C; white, H; green, D; red, O; cyan, Zn.

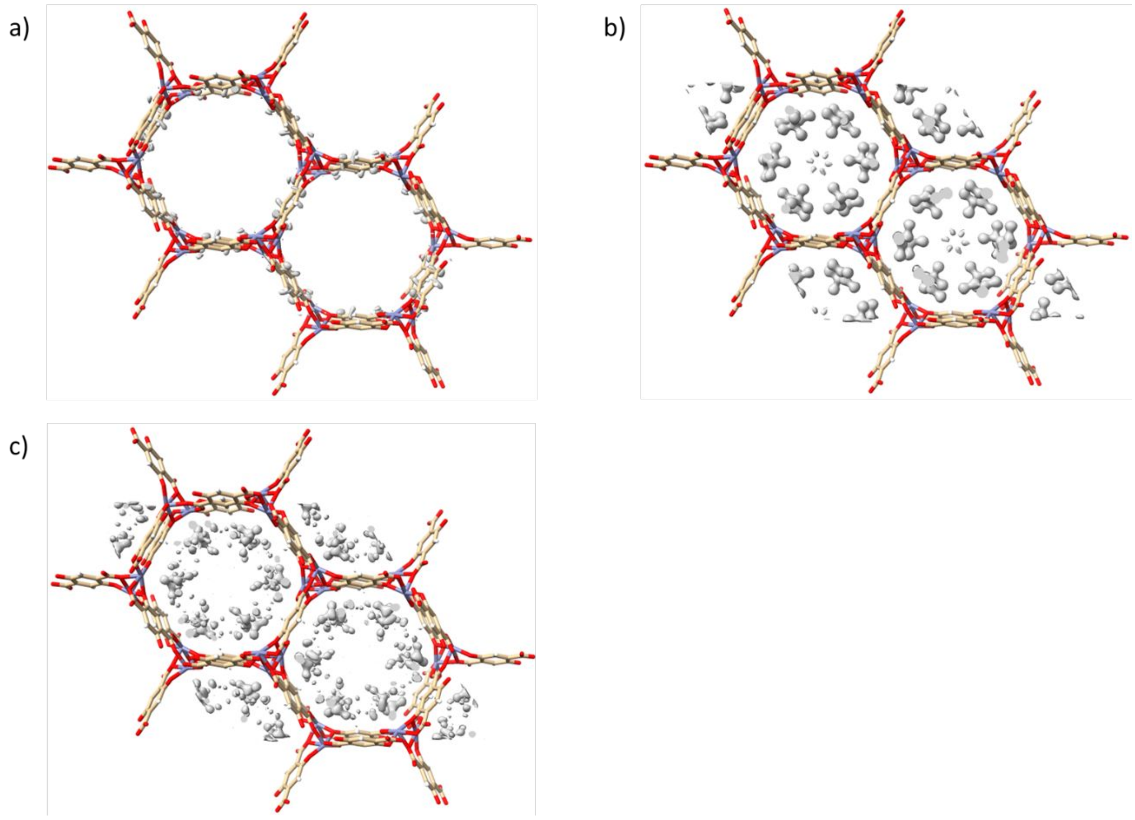

Figure S17: Difference Fourier maps (with F_{calc} of the framework model subtracted from F_{obs}) of Zn-MOF-74-0 (a), Zn-MOF-74-2 (b), and Zn-MOF-74-4 (c). All structures were measured at 226 K, only positive lobes are shown.

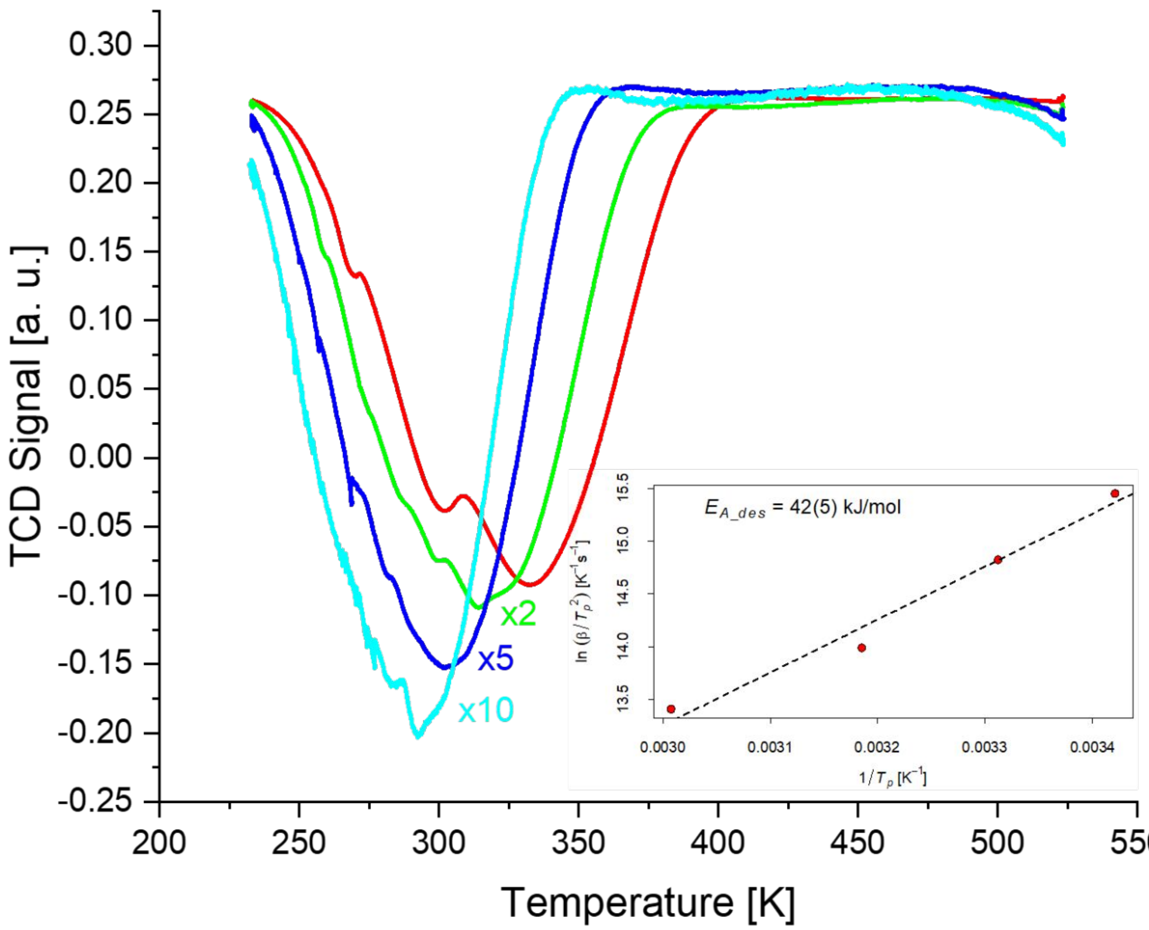

Figure S18: TPD experiments of propene-charged Zn-MOF-74. The TCD signal was recorded as a function of time, requiring the normalization by 10/β.[1, 2] The colors correspond to different heating rates: light blue (1 K/min), dark blue (2 K/min), green (5 K/ min), and red (10 K/ min). In the inset, ln(β /T_p^2) versus 1/T_p is plotted, such that the activation energy of desorption can be deduced from the slope as 42(5) kJ/mol.

From this set of experiments, the activation energy of desorption (E_{A_des}) from the OMS was determined. However, this method is not suitable for the determination of E_{A_des} of the additional two adsorption sites. It can be shown that the temperature of a peak maximum T_p of a first-order desorption process follows:

$$ln\left(\frac{\beta}{T_p^2}\right) = ln\left(\frac{A\,R}{E_{A\_des}}\right) - \frac{E_{A\_des}}{R}\left(\frac{1}{T_p}\right) \#(1)$$

Where *A* is the preexponential factor and *R* is the universal gas constant. Therefore, plotting ln(β/T_p^2) versus 1/T_p yields a linear function where the slope corresponds to E_{A_des}.[3] To obtain reasonable results, β must be varied over at least one order of magnitude.[3] It is important to note that (1) is only valid if the site coverage is identical for all experiments and the peak maximum coverage is independent of β. These assumptions are reasonable for most desorption processes performed on saturated materials.[1]

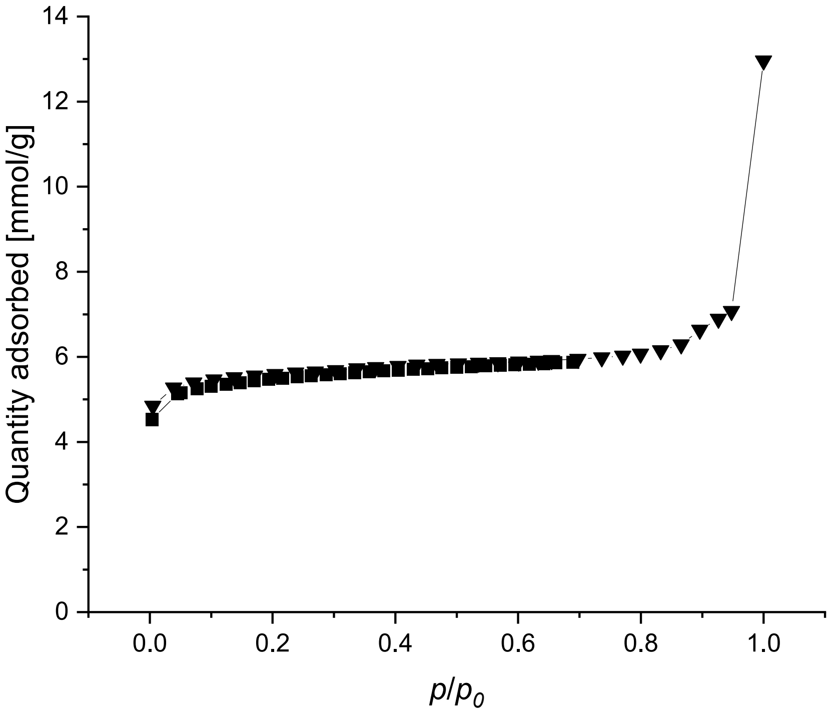

Figure S19: Physisorption of propene at 215 K with squares and triangles corresponding to the adsorption and desorption branch, respectively.

References

(1) Falconer, J. L.; Schwarz, J. A. Temperature-Programmed Desorption and Reaction: Applications to Supported Catalysts. *Cat. Rev.* **2007**, *25* (2), 141-227. DOI: 10.1080/01614948308079666.

(2) de Jong, A. M.; Niemantsverdriet, J. W. Thermal Desorption Analysis: Comparative Test of Ten Commonly Applied Procedures. *Surf. Sci.* **1990**, *233* (3), 355-365. DOI: 10.1016/0039-6028(90)90649-S.

(3) Barrie, P. J. Analysis of Temperature Programmed Desorption (TPD) Data for the Characterisation of Catalysts Containing a Distribution of Adsorption Sites. *Phys. Chem. Chem. Phys.* **2008**, *10* (12), 1688-1696. DOI: 10.1039/b717430f.
